# The first step in developing an International Classification of Functioning, Disability and Health Core Set for Vision Loss: A systematic review

**DOI:** 10.1111/opo.13269

**Published:** 2024-01-22

**Authors:** Lorenzo Billiet, Ruth M. A. van Nispen, Stijn De Baets, Ralph de Vries, Dominique Van de Velde, Hilde P. A. van der Aa

**Affiliations:** 1 Ophthalmology, Amsterdam UMC, Vrije Universiteit, De Boelelaan 1117, Amsterdam, The Netherlands; 2 Amsterdam Public Health, Quality of Care, Amsterdam, The Netherlands; 3 Faculty of Medicine and Health Sciences, Department of Rehabilitation Sciences – Occupational Therapy Research Group, Ghent University, Ghent, Belgium; 4 Blindenzorg Licht en Liefde, Varsenare, Belgium; 5 Medical Library, Vrije Universiteit, Amsterdam, The Netherlands; 6 The Lighthouse Guild, New York, New York USA

**Keywords:** activities, disability and health, ICF, ICF Core Set, International Classification of Functioning, participation, vision loss

## Abstract

**Aim:**

As a first step in developing an International Classification of Functioning, Disability and Health (ICF) Core Set for adults with vision loss, this systematic review sought to identify the researchers' perspective by identifying the most often used outcome measures and research topics obtained from studies on adults with vision loss.

**Methods:**

PubMed, Embase, CINAHL, APA PsycINFO and Web of Science were searched for studies on vision loss. Meaningful outcome measures and research topics were linked to the ICF components: environmental factors, body functions, body structures and the Activities and Participation life domains.

**Results:**

After deduplication, 7219 records remained, of which 2328 articles were eligible for further review. For feasibility reasons, approximately 20% were randomly chosen from every publication year, resulting in 446 included articles. After full-text reading, 349 articles remained, describing 753 outcome measures based on questionnaires and 2771 additional research topics that could be linked to the ICF. Most were linked to the component Activities and Participation, with a focus on recreation and leisure activities (ICF code d920, 70%), reading (d166, 34%) and driving (d475, 27%). For the component body function, seeing functions (b210, 83%) were most often reported. Outcome measures and research topics were least often linked to the body structure component and environmental factors.

**Conclusion:**

The broad range of ICF categories identified in this systematic review represents the variety of functioning typical for adults with vision loss. These results reflect the focus of researchers over the past 21 years by using various vision-related outcomes. In our next steps to develop the ICF Core Set for Vision Loss, we will include perspectives of experts and lived experience.

**Supplementary Information:**

The online version of this article (doi:10.1111/opo.13269) contains supplementary material, which is available to authorized users.

## Key points

- The outcome measures used on adults with vision loss were identified, to examine their content and demonstrate the most often studied areas of functioning and disability in this population.
- This is a first step in the creation of the International Classification of Functioning, Disability and Health Core Set Vision Loss.
- The broad range of International Classification of Functioning, Disability and Health categories identified in this systematic review represents the variety of functioning typical to adults with vision loss.

## INTRODUCTION

The International Classification of Functioning, Disability and Health (ICF) was endorsed in 2001 by the World Health Organization (WHO) and entails a classification system that not only looks at the health condition but also recognises the role of the environment in the creation of a disability. The ICF is an internationally accepted standard for describing functioning and disability with a complex interaction between a person's health condition and contextual factors. There is a continuing interaction between the different components.^1^

The first component focusses on body functions, the anatomical parts and the physiological functions and systems. The second component ‘activities and participation’ focusses on the execution of a task or action by an individual (activities) and on one's involvement in a life situation (participation). Contextual factors are the third component and focus on the personal, physical, social and attitudinal environment in which people live. Finally, personal factors of an individual, the fourth component, are not part of the health condition but entail, for instance, age, sex or coping style.^1^

The ICF is organised by over 1400 alphanumerical categories to describe all areas of functioning in an everyday clinical setting, which is far too extensive for use in practice. A solution to overcome this barrier and improve implementation is the development of condition-specific ICF Core Sets, which are limited sets of categories that are relevant for specific conditions. An ICF Core Set can facilitate the description of the functioning of individuals with a specific health condition in everyday practice by providing a comprehensive list of essential categories selected from the entire ICF. Since it is not possible to work with all the categories that have been described in the ICF, core sets have been developed, comprising a limited set of categories that are relevant for vision loss. The added value from an ICF Core Set for Vision Loss is to improve research capacity and treatment outcomes, as well as policy, to a higher level as they will meet the themes that are indicated as most relevant to patients and healthcare practitioners (e.g., social worker, low-vision therapist, medical doctor or occupational therapist) around the world, in low-, middle- and high-income countries. In addition, the Core Set can contribute to the development of standardised evaluation instruments, treatment guidelines and the mapping of areas of improvement for quality of life. The Core Set can be adapted to various assessment tools, such as questionnaires, rating systems, general interviews or observation scales, depending on the needs and wishes of the user. Moreover, it allows for comparison of data between disciplines and countries.^2–4^

To date, an ICF Core Set for people with vision loss is missing, while vision loss has a major impact worldwide. From a global perspective, in 2015, an estimated 36 million people were blind and 200 million people had a moderate to severe visual impairment. People over the age of 50 generate the largest societal burden of vision impairment.^5^ Among persons who are blind, it has been estimated that 58% are 60 years or older and 32% are between 45 and 59 years.^6^ The study of Flaxman et al.^7^ has shown that the age-specific prevalence has declined between 1990 and 2015, but absolute numbers have increased due to ageing and global population growth. In 2015, the main causes of blindness were cataract, glaucoma and age-related macular degeneration. The distribution of eye conditions as the main cause for vision loss varies across regions, income levels and ages. Researchers have observed that 90% of the individuals with visual impairment worldwide live in low-income countries.^6,8^ The prevalence of low vision and blindness will increase over the globe as a result of shifting demographics and ageing populations, for all ethnic groups.^9,10^

Evidence suggests that vision impairment has an impact on quality of life.^11^ Vision plays an important role and leads to restrictions in all areas of life when impaired. In almost all activities related to participation in social life, such as activities of daily living,^12,13^ mobility,^14^ recreation and religion, intensive visual tasks are commonly required.^8^ Individuals with visual impairment are at risk of disability, poor health, unemployment, low financial income and adverse interpersonal events.^15^ Well-known additional health problems in persons with vision loss are anxiety, depression,^16^ fatigue^17^ and bone fractures after a fall.^18–20^ Research has shown that these problems are experienced not only by older adults but also by young people with vision loss.^21^ Moreover, visual impairment is not only a problem for the individual but also a global public health problem that leads to a variety of social and economic problems.^22^

Following the methodology described by Selb et al.^23^ and used in other Core Set developments, the aim of the present study was to perform a systematic review to identify and quantify variables commonly addressed in published studies focussing on the functioning and disability of persons with vision loss. In the creation of the ICF Core Set for Vision Loss and as a first step to describe the researchers' perspective, the variables obtained from outcome measures and research topics will be mapped to the conceptual framework of the ICF. These variables reflect the frequency with which different outcomes have been used, which is influenced by researchers' views on what is important to the field, as well as the standard outcome measures available.

## METHODS

To conduct the systematic review, we followed the Preferred Reporting Items for Systematic Reviews and Meta-Analyses (PRISMA) guidelines.^24^ We used a predetermined methodology that makes it possible to follow a sufficiently evidence-based process in the development of the ICF Core Set.^23^ In preparation, a protocol paper combined with an invitation for organisations worldwide to collaborate has been published.^25^

### Inclusion and exclusion criteria

The inclusion criteria for the systematic review were (1) the *study population* was referred to as adult (≥18 years old); (2) the study population experienced vision loss varying from mild to blindness as defined by the World Health Organization: a distance visual impairment varying from mild (presenting visual acuity worse than 6/12 in the better eye), moderate (between 6/18–6/60 in the better eye), severe (between 6/60–3/60) to blindness (worse than 3/60 or no light perception), and near visual impairment was defined as acuity worse than N6 or M0.8 at 40 cm and best-corrected visual acuity equal to or worse than 6/12; (3) visual field loss, defined as hemianopsia, constriction to <50°, loss of 50% of central 30° was taken into account; (4) different vision conditions were allowed as long as they were not part of another medical condition (e.g., visual impairment as a result of a brain tumour); (5) the *sample size* of the included research was ≥10 subjects; (6) English was the publication language and (7) various types of *study designs* were included: systematic reviews, observational studies, prospective longitudinal studies, randomised controlled trials, cross-sectional studies, cohort studies and (8) psychometric studies. No distinction was made between qualitative and quantitative studies.

The exclusion criteria were (1) non-human populations; (2) publication date before the introduction of the ICF in 2001; (3) studies that focussed on evaluating prevalence estimates and interventions (e.g., surgery, medical studies or cost-effectiveness studies) and (4) studies where the primary health condition of the target group was not vision-related or where visual impairment was a comorbid condition (e.g., Usher syndrome).^26^

### Database search

To identify relevant publications, we conducted a systematic search in different bibliographic databases (i.e., PubMed, Embase.com, CINAHL, APA PsycINFO and Web of Science) from 2001 to March 2022. The work was done in collaboration with a medical information specialist with experience in conducting database searches. The scientific literature was examined to find appropriate search terms. During the search, standardised search terms were used (i.e., MeSH or Emtree) combined with Boolean expressions. Some search terms were added after reading the first sample of articles and analysing the study or search strings used to develop other Core Sets.^27–29^

The database search was based on three clusters of search terms (including synonyms and closely related words) as index terms or free-text words: (1) the study population (all terms related to vision loss and eye diseases), (2) outcome measures and (3) activities of daily living and quality of life. The exclusion criteria filter was used for sample size, age, language and study design. Duplicate articles were excluded. The complete search strings for all databases are presented in Appendix A in Supporting Information.

### Reviewing a sample of the papers

Due to the overwhelming number of studies published in the area of vision loss, the procedure recommended by Selb et al. was followed, which states that for feasibility reasons only a sample (i.e., approximately 10%) of the identified papers needs to be reviewed and analysed. Previous systematic reviews that also were performed with the aim of developing an ICF Core Set followed the same procedure.^23^

### Linking procedure

In the last step of the systematic review, all articles were read and meaningful concepts linked to the most specific ICF categories using standardised linking rules. Outcome measures based on questionnaires and other research topics were examined.

The ICF is organised hierarchically: the domains are followed by chapters (first digit of the alphanumeric code), and each chapter consists of second-level categories, the second and third digit. These are followed by more detailed third- and fourth-level categories as shown in the following example from the component activities and participation (d): (1) first-level chapter: d4,‘Mobility’; (2) second-level category: d475, ‘Driving’; and (3) third-level category: d4751 ‘Driving motorised vehicles’.

When linking data to the ICF, standardised linking rules were used.^30^ The first key points of the linking rules was that meaningful concepts have to be linked to the most precise ICF category in the component of body functions (denoted by the letter ‘b’), body structures (s), activities and participation (d) and environmental factors (e). Personal factors (pf) were also linked to the ICF, although they have not yet been classified in the ICF. The second key factor is that the Code 8 (otherwise specified) or 9 (unspecified) was only used when there was no other option. In this study, the code not definable (nd) was not used. The third key point was that only one category was chosen for linking a meaningful concept, while the fourth was that if an ICF category was repeatedly assigned within one multiple-item measure or within one study, it was counted only once.

From each included study, information was extracted and frequency analysis was used to examine the total number of outcome measures. The decision whether a particular meaningful concept was included in the Core Set depended on how often it occurred. Subsequently, ICF categories were linked along with corresponding percentages relative to the number of studies in which they were used. ICF categories found in at least 5% of the studies were included in the list of candidate categories. To be consistent with previous ICF Core Set studies, the second-level categories were reported in the temporary Core Set. Concepts associated with a more specific third- or fourth-level category were reported in their corresponding second-level category.^28^

## RESULTS

### Selection process

**FIGURE 1:**
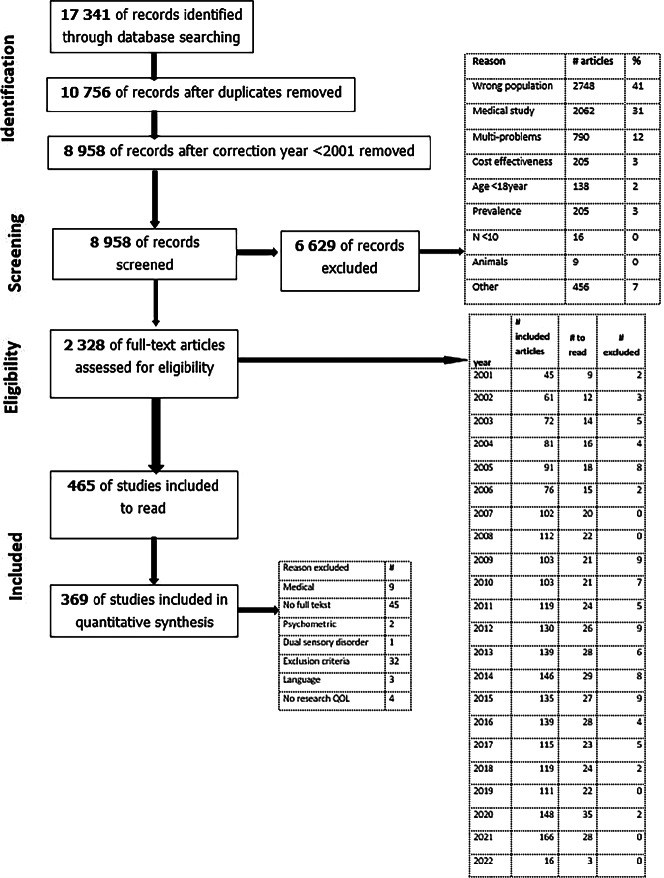
Flow diagram of the literature review.^24^

The literature search generated a total of 17,341 references: 4754 in PubMed, 6043 in Embase, 2115 in CINAHL, 657 in PsycINFO and 3772 in Web of Science. Results from the searches were imported in EndNote and duplicates were removed. This resulted in 10,756 articles. Since the introduction from the ICF was in May 2001, a correction in the search outcomes was made, which resulted in 8958 articles (Figure 1).

Due to the overwhelming number of studies, the procedure recommended by Selb et al.^23^ was followed, and only the first 10% of all potentially relevant titles and abstracts were reviewed, spread equally over all publication years. In this first round, titles and abstracts until March 2022 were screened for eligibility by two independent reviewers (BL and vNRMA). In 44 cases, there was doubt about the inclusion and exclusion criteria. Differences in judgement were resolved through discussion. The screening with the two independent reviewers corresponded to a Cohen's kappa of 0.94, which indicates a high degree of independence and a small chance of coincidence. If there was no abstract available, then the full text of the article was checked for eligibility. Appendix B in Supporting Information shows an overview of all included articles.

After reading the 465 articles, a further 21% were excluded, resulting in 369 eligible articles. Analysis of the excluded articles indicated that the most common reason for exclusion was the absence of a full text (45 articles) and not meeting the inclusion criteria (32 articles). If the full-text article was missing, an attempt was made to request it from the authors; 19% of the requested articles were obtained this way. Adding extra articles to the review did not seem to have added value.

### Study selection and characteristics

When looking at the total number of eligible articles (*N* = 2328), there was an average of 101 (SD ± 38) publications per year between January 2001 and March 2022, with a peak in 2014 in which 146 publications were retained.

**FIGURE 2:**
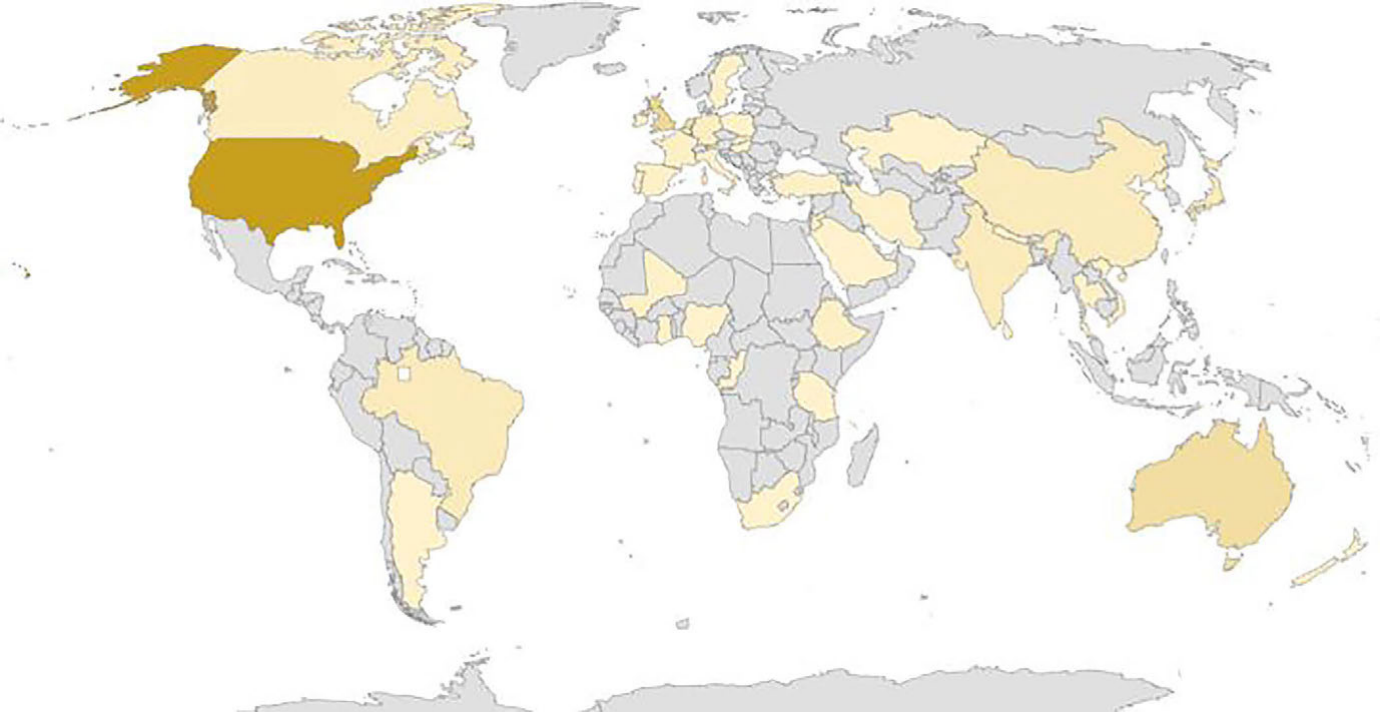
Geographic distribution of the studies linked to the International Classification of Functioning, Disability and Health (ICF). The darker the colour, the more studies come from that country.

The studies were conducted in various countries and WHO regions (Figure 2 and Table 1), that is, America (38.0%), South-East Asia (5.9%), Europe (30.6%), Eastern Mediterranean (2.2%), Western Pacific (18.5%) and African region (4.8%). In 98 articles, no regions were mentioned; this mainly concerned systematic reviews.

**TABLE 1:** Characteristics of included studies (*N* = 369).

	*N* (%)
WHO Region of the Americas	**103** (28)
Argentina	1
Brazil	9
Canada	6
United States of America	87
WHO South-East Asia Region	**16** (4.3)
Democratic People's Republic of Korea	3
India	9
Nepal	1
Sri Lanka	1
Thailand	2
WHO European Region	**83** (22.5)
Belgium	1
France	4
Germany	6
Greece	1
Hungary	1
Ireland	1
Israel	2
Italy	3
Kazakhstan	1
The Netherlands	23
Portugal	2
Spain	3
Poland	1
Slovakia	1
Sweden	3
Turkey	3
United Kingdom	27
WHO Eastern Mediterranean Region	**6** (1.6)
Iran	3
Saudi Arabia	1
Jordan	2
WHO Western Pacific Region	**50** (13.5)
Australia	22
China	9
Japan	9
Malaysia	4
New Zealand	1
Singapore	3
Vietnam	2
WHO African Region	**13** (3.5)
Ethiopia	4
Ghana	3
Mali	2
Nigeria	1
Congo	1
South Africa	1
Tanzania	1
No regions	98 (26.5)

Abbreviation: WHO, World Health Organization.

*Note*: The total for each WHO region is shown in bold.

The total sample sizes of the included articles (*N* = 369) ranged from 10 to 47,527 participants (median 145.5, interquartile range 57–449.5). In 204 of the articles (55%), the study was conducted with persons with a specific eye condition. A total of 36 different ocular disorders were identified in this systematic review. The most common diagnoses studied were glaucoma (28%), followed by macular degeneration (25%) and diabetic retinopathy (10%).

### Linking the outcome measures

First, meaningful concepts were analysed by looking at the outcome measurements used in research and linked the content of these outcome measurements to the ICF.

A variety of questionnaires and outcome measurements were used in the various studies. Across all analysed articles, 100 different questionnaires or outcome measurements were used to map human functioning (Appendix C in Supporting Information). After linking all meaningful concepts to the ICF, there were a total of 753 constructs. The majority of all measured constructs were linked to the component activities and participation (72%), a minority of 20% to body functions and 8% to environmental factors.

Overall, six concepts (0.8%) were linked to a first-level ICF category, 62 concepts (8.2%) to a second-level category, 240 (31.8%) to a third-level category and 445 (59.1%) to a fourth-level ICF category (see Table 2).

**TABLE 2:** Distribution of the measurement outcomes over the different chapters in the International Classification of Functioning, Disability and Health (ICF) (*n* = 753).

	1st level	2nd level	3rd level	4th level	*N* (%)
s					0 (0)
b	1	6	4	140	151 (20)
d	5	20	215	305	545 (72)
e		36	21		57 (8)
*N*	6	62	240	445	
%	0.80	8.23	31.8	59.1	

*Note*: Empty boxes indicate no frequencies. *N* = the total number of meaningful concepts that were linked to the ICF.

Abbreviations: b, body functions; d, activities and participation; e, environmental factors; s, body structures.

In the component body functions, there was one meaningful concept linked to the first level, six to the second level, four to the third level and 140 to the fourth level. The most common categories within the component body functions were light sensitivity (b21020, in 20% of all outcome measurements), regulation of emotions (b1521, 20%), energy level (b1300, 16%) and sustaining attention (b1400, 9%). ‘Activities and participation’ was the most frequently covered component, including five meaningful concepts at first-level category, 20 at second-level category, 215 at third-level category and 305 at fourth-level category. Of all linked constructs, there were 30% in the chapter mobility (d4) with the rubric moving around outside the home and other buildings (d4602, 20%) and climbing stairs (d451, 27%). The most common categories within the Activities and Participation component were the chapters learning and applying knowledge (d1, mainly watching and reading), and community, social and civic life (d9, recreation and leisure). In 24% of the cases, constructs were found in the chapters self-care (d5) and household (d6). Important categories in the component environmental factors were friends, (e320, 11%), immediate family (e310, 9%) and light (e240, 7%) (see Table 3 and Figure 5).

**TABLE 3:** An overview of the absolute frequencies linked to the International Classification of Functioning, Disability and Health (ICF) from the analysed outcome measurements used in research.

Life domains	ICF code	ICF category code description	Measured in number of outcome measurements (%)
	**b**	**Body functions**	
Mental functions	b1300	Energy level	16
	b1340	Amount of sleep	7
	b1341	Onset of sleep	4
	b1343	Quality of sleep	5
	b1400	Sustaining attention	9
	b1521	Regulation of emotion	20
	b1801	Body image	8
Sensory functions and pain	b21020	Light sensitivity	20
	b21021	Colour vision	8
	b280	Pain	5
		**d**	**Activities and participation**
Learning and applying knowledge	d110	Watching	16
	d130	Copying	5
	d166	Reading	28
	d170	Writing	8
Communication	d3150	Communicating with receiving body gestures	16
	d3151	Communicating with receiving—general signs and symbols	14
Mobility	d4500	Walking short distances	6
	d4502	Walking on different surfaces	4
	d4503	Walking around obstacles	15
	d451	Climbing	27
	d460	Moving around in different locations	4
	d4600	Moving around within the home	12
	d4601	Moving around within buildings other than home	5
	d4602	Moving around outside the home and other buildings	20
	d465	Moving around using equipment	4
	d4702	Using public motorised transportation	11
	d475	Driving	11
	d4750	Driving human-powered transportation	4
	d4751	Driving motorised vehicles	4
Self-care	d510	Washing oneself	6
	d5202	Caring for hair	5
	d5203	Caring for fingernails	5
	d5400	Putting on clothes	7
	d5404	Choosing appropriate clothing	6
	d550	Eating	10
	d560	Drinking	5
	d5702	Maintaining one's health	7
Domestic life	d6200	Shopping	11
	d630	Preparing meals	11
	d640	Doing housework	7
	d6400	Washing and drying clothes and garments	4
	d6402	Cleaning living area	6
	d650	Caring for household objects	5
	d6500	Making and repairing clothes	5
Interpersonal interactions and relationships	d730	Relating with strangers	5
	d7500	Friends	9
	d7702	Sexual relationship	7
Major life areas	d810	Education	7
	d840	Work and employment	7
	d860	Basic economic transaction	6
	d865	Complex economic transaction	4
	d8700	Personal economic resources	4
Community, social and civic life	d920	Recreation and leisure	9
	d9200	Play	9
	d9201	Sports	11
	d9202	Arts and culture	9
	d9204	Hobbies	6
	d9205	Socialising	14
	**e**	**Environmental factors**	
Products and technology	e1150	General products and technology for personal use in daily living	5
Natural environment and human-made changes to environment	e240	Light	7
Support and relationships	e310	Immediate family	9
	e320	Friends	11

**FIGURE 3:**
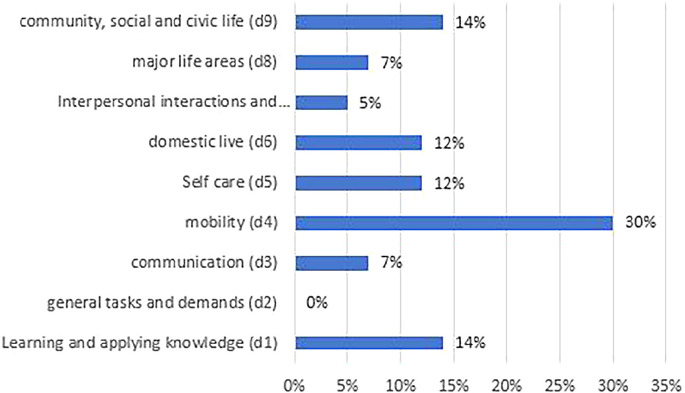
Distribution of constructs over the component activities and participation.

The most common constructs linked to the ICF were found in the category mobility (30%), followed by the chapters learning and applying knowledge (14%), and community, social and civic life (14%) (see Figure 3).

### Linking the research topics

In the second step, all of the topics and content that were investigated or mentioned in research studies were examined and the content was linked to the ICF. This linking differs from the linking of the outcome measures because the focus was on the topic that was studied and not the content of the complete outcome measures.

Overall, 2771 concepts were linked to an ICF component. Most of the concepts were linked on the third and fourth level: 0.83% of concepts could only be linked to a first-level category, 0.83% to the second level, 33.5% to the third and most concepts (64.81%) were on the fourth-level ICF categories. In total, 108 concepts were linked to the code ‘unspecified’ (these are items whose ICF code ends with 9), in 11% of the cases to body functions, 84% to activities and participation and 2% to environmental factors. The most often covered component was Activities and Participation with 17 meaningful concepts at the second level, 623 at third level and 1064 at fourth level (Table 4).

**TABLE 4:** Distribution over the linked articles (*n* = 2771).

	1st level	2nd level	3rd level	4th level	*n* (%)
s			7	2	9 (0)
b		3	120	548	671 (24)
d	19	17	623	1064	1723 (62)
e	4	3	179	182	368 (13)
*N*	23	23	929	1796	
%	0.83	0.83	33.53	64.81	

*Note*: Empty boxes indicate no frequencies. *N* = the total number of meaningful concepts that were linked to the International Classification of Functioning, Disability and Health (ICF).

Abbreviations: b, body functions; d, activities and participation; e, environmental factors; s, body structures.

In Table 5, an overview is presented for the categories on the second level linked to the ICF. When we took a closer look at the component body functions, 83% of all articles were linked to seeing functions (b210) and in 24% of the cases, there was a link with emotional functions (b152). For the component activities and participation, 70% of the meaningful concepts in research articles were linked to recreation and leisure (d920), 34% of all articles were linked to reading (d166) and 27% to driving (d475). In the component environmental factors, products and technology for personal use in daily life (e115, 15%), products and technology for communication (e125, 9%) and the presence of immediate family (e310, 9%) were linked.

**TABLE 5:** An overview of the relative frequencies of second-level categories identified and linked to the International Classification of Functioning, Disability and Health (ICF).

Life domains	ICF code	ICF category code description	Used in number of studies (*n*/%^a^)	
	**b**	**Body functions**		
Mental functions	b114	Orientation functions	29	8%
	b126	Temperament and personality functions	23	6%
	b152	Emotional functions	95	26%
	b156	Perceptual functions	20	5%
Sensory functions and pain	b210	Seeing functions	306	83%
	b240	Sensation associated with hearing and vestibular function	27	7%
	b280	Sensation of pain	27	7%
	**d**	**Activities and participation**		
Learning and applying knowledge	d110	Watching	42	11%
	d170	Writing	31	8%
General tasks and demands	d230	Carrying out daily routine	26	7%
	d240	Handling stress and other psychological demands	19	5%
Communication	d315	Non-verbal messages	57	15%
	d360	Using communication devices and techniques	42	11%
Mobility	d450	Walking	83	22%
	d460	Moving around in different locations	83	22%
	d470	Using transportation	22	6%
	d475	Driving	100	27%
Self-care	d540	Dressing	32	9%
	d570	Looking after one's health	42	11%
Domestic life	d620	Acquisition of goods and services	52	14%
	d630	Preparing meals	40	11%
	d640	Doing housework	49	13%
	d650	Caring for household objects	25	7%
Interpersonal interactions and relationships	d750	Informal social relationship	40	11%
	d770	Intimate relationship	22	6%
Major life areas	d845	Acquiring, keeping and terminating a job	27	7%
	d850	Remunerative employment	22	6%
	d860	Basic economic transaction	30	8%
	d865	Complex economic transaction	25	7%
Community, social and civic life	d920	Recreation and leisure	259	70%
	d930	Religion and spirituality	21	6%
	**e**	**Environmental factors**		
Products and technology	e115	Products and technology for personal use in daily living	57	15%
	e120	Products and technology for personal indoor and outdoor mobility and transportation	27	7%
	e125	Products and technology for communication	34	9%
Natural environment and human-made changes to environment	e240	Light	22	6%
Support and relationships	e310	Immediate family	34	9%
	e320	Friends	29	8%

^a^From the number of studies included in the systematic review.

When looking closely at the components on the third and fourth levels in the body functions orientation to place (b1141), regulating emotions (b1521) and more specifically depressed feelings and fears were often linked. Pain was also often linked, and more specifically ocular pain (b28010).

**FIGURE 4:**
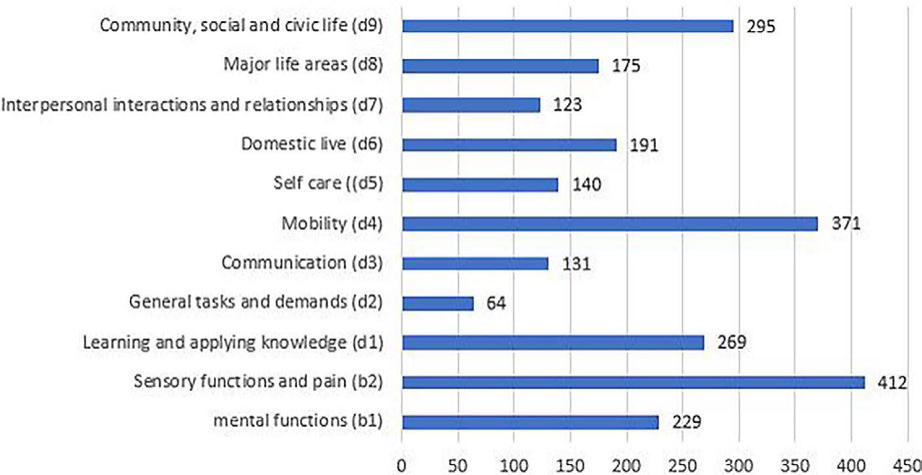
Distribution of linking meaningful concepts across the different chapters (absolute frequencies).

The activities and participation component accounts for 63.5% of all linked meaningful concepts. There was a distribution over the different chapters, with outliers for mobility and community, social and civic life. In the domain of recreation and leisure, 43% of the meaningful concepts were linked to ‘socialising’ (d9205), 15% to ‘sports’ (d9201) and 13% to ‘exercising one's own hobby’ (d9204) (Figure 4).

In 30% of the studies, mobility was identified as a meaningful concept. Of these, 10% concerned driving (21% driving motorised vehicles, d4751), 22% moving around in different locations (d460) and 16% the use of transportation; this mainly referred to the utilisation of public motorised transportation (d4702) and using humans for transportation (d4703).

The third most often linked component was reading (d166), which accounted for a meaningful concept in 124 selected articles. It was often linked to problems with reading signs and symbols (d3151).

**FIGURE 5:**
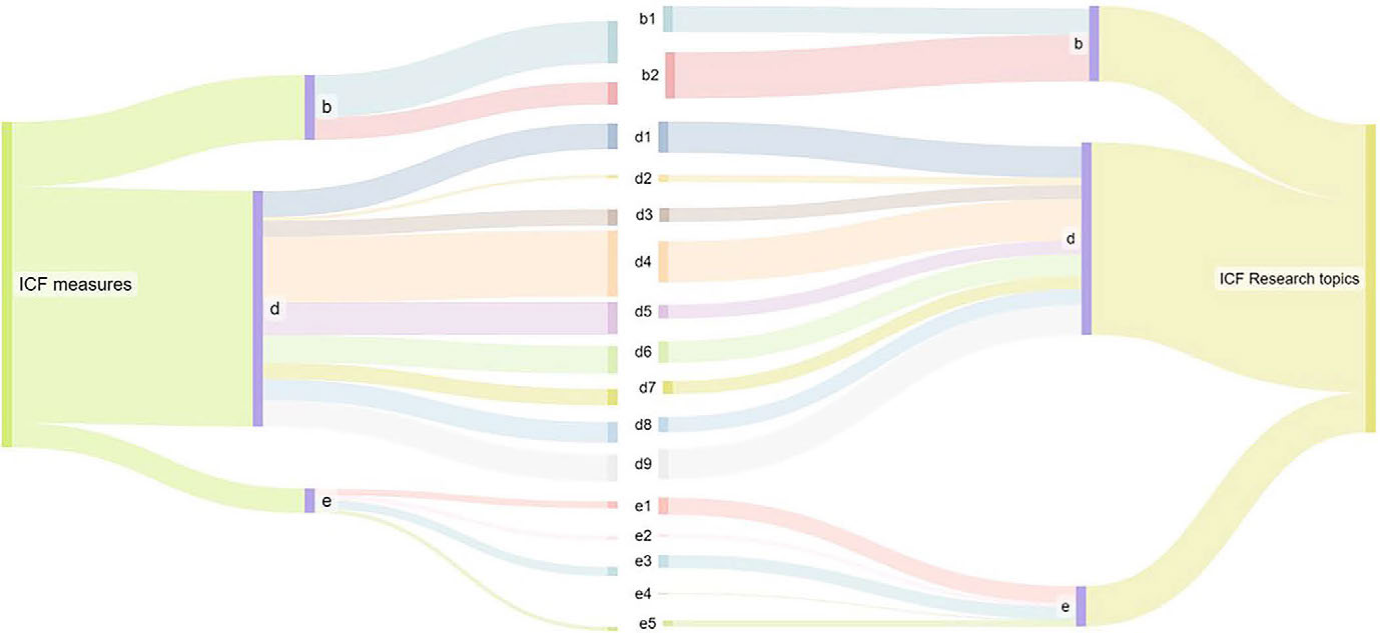
Overview of the linking where the outcome measurements were compared with the research topics. ICF, International Classification of Functioning, Disability and Health. Abbreviations: b, body functions; d, activities and participation; e, environmental factors; s, body structures.

When comparing the results of the outcome measures and the research topics analysed, no significant differences were observed. Research topics indicated a growing emphasis on the natural environment and human-made changes to the environment (e4) (Figure 5).

## DISCUSSION

This systematic review is the first to provide an overview of the most studied research topics, and used outcome measures in the field of low vision over the past two decades. This identified researchers' perspective about what is important when examining functioning in individuals with vision loss in an ICF Core Set.

The results showed that many studies concentrated on the component ‘body functions”. Important chapters of body functions were orientation to place, regulating emotions and especially depressed mood and anxiety. This is consistent with the literature in which a relation between visual impairment and depression, fatigue and anxiety has been reported.^16,17,31^ For the seeing function, four important factors emerged, namely visual field functions, light sensitivity, colour vision and contrast sensitivity.

Another important component was ‘Activities and Participation’. When linking meaningful chapters within the Activities and Participation component, the three most often studied outcomes were reading, mobility, and recreation and leisure, which might reflect the topics most affected by severe vision loss, leading to loss of independence. The notion that vision loss is associated with a decline in mobility is well known. For example, in glaucoma or retinitis pigmentosa, the visual field narrows, and bumping into obstacles and a slower walking pace are often reported.^32^

A third important component was ‘environmental factors’, indicating the ability to move independently through the environment. Social and spatial issues such as inaccessible transportation or buildings and the fear of falling were important barriers in mobility for persons with vision loss.^14,33,34^ When looking closer at the environmental factors, support from family and friends were often mentioned topics. Bassey and Ellison^35^ found that participants experienced reduced social contacts and negative social support from friends, colleagues and employers, but more social contacts and positive social support from family members. As a result, people may experience feelings of loneliness.^36^ Based on our sample, less research seems to have been conducted on the role and the grade in which formal and informal social support was provided.

When items from measurement instruments were linked to the ICF, remarkably, no items or constructs were found that could be linked to the Activities and Participation life domain ‘general tasks and demands’. In this domain, various topics were addressed such as carrying out a daily routine, handling stress and undertaking single and multiple tasks. This indicates that the measurement instruments in this sample do not measure this topic, which is important for people with vision loss.

To develop the ICF Core Set for Vision Loss, follow-up studies will be performed in which the researchers' perspective observed in this systematic review will be placed against those that exist among patients, healthcare providers and other experts within the field of vision loss. Together, this constitutes the preparatory phase of developing the ICF Core Set for Vision Loss and captures the complete perspectives of researchers, patients, healthcare providers and other experts. The information from this preparatory phase will be brought together and discussed in a consensus conference, in which all stakeholders come together to develop a final internationally accepted ICF Core Set for Vision Loss. This Core Set will provide a practical tool that maps the entire spectrum of functioning. We aim to provide a common language that can be used to document, report and assess vision loss performance across the globe.

## STRENGTHS AND LIMITATIONS

The strength of this research lies in the fact that several databases were used with carefully selected keywords, chosen in collaboration with an experienced medical information specialist. The systematic review was established by following a frequently used methodological approach. Highly experienced researchers from the fields of low vision and rehabilitation were involved in this process, and their advice was sought in making final decisions.

However, this study also has certain limitations that need to be considered when interpreting the results. In the search strategy, specific terms that referred to body functions or environmental factors were not included. This may have led to an underrepresentation of outcome measures and research topics related to these concepts. Specific attention should be paid to these concepts in the follow-up research to complete the Core Set Vision Loss. Another limitation is the possibility that the same article was included twice and therefore a double analysis of the same article was performed. This may have occurred if the article was included in a review and in parallel, the original study was also included.

Selected studies often focussed on glaucoma, which may have introduced a potential limitation to the generalisability of our findings, although glaucoma has been a prominent area of study in the Global South, while macular degeneration is more prevalent in the Global North region.^6,7^ The prevalence and impact of vision-related conditions may vary across regions. Therefore, it is imperative to maintain a global outlook. The present study may not fully reflect the impact of vision loss on functioning, as it could be studied more often in the context of the Global South and North. This limitation could reflect a bias in the random selection of studies included here.

In our aim to develop a culture and context neutral Core Set, there seems to be an underrepresentation of the African region and Eastern Mediterranean Sea area, which should be considered in the next steps of the Core Set development.

## CONCLUSION

This systematic review identified the outcome measures used in studies of adults with vision loss, to examine their content and demonstrate the most often studied areas of functioning and disability in this population. For the analyses of the researchers perspective, a vast amount of literature was examined and most of the topics on the component ‘Activities and Participation’ could be linked to (1) mobility, (2) learning and application of knowledge and (3) community, social and civic life. Within the component of ‘body functions’, much attention was paid to the seeing functions and emotions. Within environmental factors, friends, family and lighting were important and much attention was directed to (digital) products and technology for personal use in daily life. The ICF Core Set for Vision Loss should focus on these aspects to determine the variety of functioning typical to people with visual impairment. The Core Set Vision Loss is potentially important to the field because it can play a critical role in improving policies, research and treatment outcomes by addressing issues most relevant to patients and healthcare providers. In addition, it lays the foundation for standardised evaluation tools, documents, treatment guidelines or the identification of areas for improvement in the quality of life and makes it possible to compare data across disciplines and countries.

In the next steps, the perspectives of patients and healthcare professionals on important topics that should be part of the ICF Core Set for Vision Loss will be addressed. This will allow mapping of the entire spectrum of functioning for adults with visual impairment.

## Supplementary Information

Supplementary file (DOCX 77.2 KB)
