# Supplementary material for: The first step in developing an International Classification of Functioning, Disability and Health Core Set for Vision Loss: A systematic review
Source: Ophthalmic Physiol Opt. 2024 Jan 22;44(2):413–25. doi: 10.1111/opo.13269 (PMC12872645; doi:10.1111/opo.13269)
Supplement: Supplementary file 1 — Supplementary file (DOCX 77.2 KB) [file 44402_2024_4402017_MOESM1_ESM.docx]

## Supplementary material: Appendix A

**PubMed Session Results (27 Jan 2022)**

| Search | Query | Items found |
| --- | --- | --- |
| #5 | #4 NOT (("Adolescent"[Mesh] OR "Child"[Mesh] OR "Infant"[Mesh] OR adolescen*[tiab] OR child*[tiab] OR schoolchild*[tiab] OR infant*[tiab] OR girl*[tiab] OR boy[tiab] OR boys[tiab] OR teen[tiab] OR teens[tiab] OR teenager*[tiab] OR youth*[tiab] OR pediatr*[tiab] OR paediatr*[tiab] OR puber*[tiab]) NOT ("Adult"[Mesh] OR adult*[tiab] OR man[tiab] OR men[tiab] OR woman[tiab] OR women[tiab])) | 4,754 |
| #4 | #1 AND #2 AND #3 | 5,128 |
| #3 | "Quality of Life"[Mesh] OR "quality of life"[tiab] OR "life qualit*"[tiab] OR "living qualit*"[tiab] OR "quality of living"[tiab] OR "Activities of Daily Living"[Mesh] OR "activities of daily living"[tiab] OR "activity of daily living"[tiab] OR "activities of daily life"[tiab] OR "activity of daily life"[tiab] OR "daily living activit*"[tiab] OR "daily life activit*"[tiab] OR adl[tiab] OR "chronic limitation of activity"[tiab] OR "Self Care"[Mesh] OR "self care*"[tiab] OR "Health Status"[Mesh] OR "health status"[tiab] OR "level of health"[tiab] OR "health level*"[tiab] OR qol[tiab] OR hrql[tiab] OR hrqol[tiab] OR participation[tiab] OR handicap[tiab] OR productivity[tiab] OR reintegration[tiab] OR "re-integration"[tiab] | 933,897 |
| #2 | "Disability Evaluation"[Mesh] OR "International Classification of Functioning, Disability and Health"[Mesh] OR "Surveys and Questionnaires"[Mesh] OR questionnaire*[tiab] OR "outcome measure*"[tiab] OR scale*[tiab] OR survey*[tiab] | 2,791,261 |
| #1 | "Visually Impaired Persons"[Mesh] OR "Vision Disorders"[Mesh:noexp] OR "Blindness"[Mesh] OR "Vision, Low"[Mesh] OR "Retinal Diseases"[Mesh:noexp] OR "Diabetic Retinopathy"[Mesh] OR "Leber Congenital Amaurosis"[Mesh] OR "Retinal Artery Occlusion"[Mesh] OR "Retinal Degeneration"[Mesh] OR "Retinal Detachment"[Mesh] OR "Retinal Hemorrhage"[Mesh] OR "Retinal Neovascularization"[Mesh] OR "Retinal Vein Occlusion"[Mesh] OR "Vitreoretinopathy, Proliferative"[Mesh] OR "Eye Diseases"[Mesh:noexp] OR "Corneal Diseases"[Mesh] OR "Eye Diseases, Hereditary"[Mesh] OR "Ocular Hypertension"[Mesh] OR "Optic Nerve Diseases"[Mesh] OR "Hemianopsia"[Mesh] OR "visually impair*"[tiab] OR "visual impair*"[tiab] OR "vision impair*"[tiab] OR blindness*[tiab] OR "low vision*"[tiab] OR "reduced vision*"[tiab] OR "subnormal vision*"[tiab] OR "diminished vision*"[tiab] OR "vision disorder*"[tiab] OR "visual disorder*"[tiab] OR "vision disab*"[tiab] OR "visual disab*"[tiab] OR "visually disab*"[tiab] OR "retinal disease*"[tiab] OR "retina disease*"[tiab] OR "retinal disorder*"[tiab] OR "retina disorder*"[tiab] OR "diabetic retinopath*"[tiab] OR leber[tiab] OR leber's[tiab] OR lebers[tiab] OR "retinal artery occlusion*"[tiab] OR "retinal degeneration*"[tiab] OR "retina degeneration*"[tiab] OR "macular degeneration*"[tiab] OR "macula degeneration*"[tiab] OR "macular dystroph*"[tiab] OR "macula dystroph*"[tiab] OR maculopath*[tiab] OR "macular edema*"[tiab] OR "macula edema*"[tiab] OR "macular oedema*"[tiab] OR "macula oedema*"[tiab] OR "retinitis pigmentosa"[tiab] OR "Rod-Cone dystroph*"[tiab] OR "Cone-Rod dystroph*"[tiab] OR "retinal detachment*"[tiab] OR "retina detachment*"[tiab] OR "retinal hemorrhage*"[tiab] OR "retinal haemorrhage*"[tiab] OR "retinal neovascularization*"[tiab] OR "retinal neovascularisation*"[tiab] OR "retinal vein occlusion*"[tiab] OR "vitreoretinopath*"[tiab] OR "vitreo-retinopath*"[tiab] OR "corneal disease*"[tiab] OR "cornea disease*"[tiab] OR "corneal disorder*"[tiab] OR "cornea disorder*"[tiab] OR glaucoma*[tiab] OR "optic nerve disease*"[tiab] OR "optic nerve disorder*"[tiab] OR "optic neuropath*"[tiab] OR "optic atroph*"[tiab] OR hemianop*[tiab] OR quadrantanop*[tiab] OR adynamia[tiab] OR legasthenia[tiab] OR "vision loss*"[tiab] OR cataract*[tiab] | 450,839 |

**Embase.com Session Results (27 Jan 2022)**

| Search | Query | Items found |
| --- | --- | --- |
| #6 | #5 NOT ('conference abstract'/it OR 'conference review'/it) | 6,043 |
| #5 | #4 NOT (('adolescent'/exp OR 'child'/exp OR adolescent*:ti,ab OR child*:ti,ab OR schoolchild*:ti,ab OR infant*:ti,ab OR girl*:ti,ab OR boy*:ti,ab OR teen:ti,ab OR teens:ti,ab OR teenager*:ti,ab OR youth*:ti,ab OR pediatr*:ti,ab OR paediatr*:ti,ab OR puber*:ti,ab ) NOT ('adult'/exp OR 'aged'/exp OR 'middle aged'/exp OR adult*:ti,ab OR man:ti,ab OR men:ti,ab OR woman:ti,ab OR women:ti,ab)) | 8,193 |
| #4 | #1 AND #2 AND #3 | 8,835 |
| #3 | 'quality of life'/exp OR (life NEXT/1 qualit*):ab,ti,kw OR 'quality of life':ab,ti,kw OR 'daily life activity'/exp OR 'activities of daily living':ab,ti,kw OR ('daily living' NEXT/1 activit*):ab,ti,kw OR ('daily live' NEXT/1 activit*):ab,ti,kw OR 'adl':ab,ti,kw OR 'chronic limitation of activity':ab,ti,kw OR (self NEXT/1 care*):ab,ti,kw OR 'health status'/exp OR 'health status':ab,ti,kw OR 'level of health':ab,ti,kw OR (health NEXT/1 level*):ab,ti,kw OR 'qol':ab,ti,kw OR 'hrql':ab,ti,kw OR 'hrqol':ab,ti,kw OR participation:ab,ti,kw OR handicap:ab,ti,kw OR productivity:ab,ti,kw OR reintegration:ab,ti,kw OR 're-integration':ab,ti,kw | 1,330,332 |
| #2 | 'International Classification of Functioning, Disability and Health'/exp OR 'questionnaire'/exp OR 'health survey'/exp OR 'health care survey'/exp OR 'survey'/exp OR 'surveys'/exp OR questionnaire*:ab,ti,kw OR 'outcome measure*':ab,ti,kw OR scale*:ab,ti,kw OR survey*:ab,ti,kw | 3,119,231 |
| #1 | 'visually impaired person'/exp OR 'visual disorder'/de OR 'visual impairment'/exp OR 'retina disease'/de OR 'diabetic retinopathy'/exp OR 'retina artery occlusion'/exp OR 'retina degeneration'/exp OR 'retina detachment'/exp OR 'retina hemorrhage'/exp OR 'retina neovascularization'/exp OR 'retina vein occlusion'/exp OR 'vitreoretinopathy'/exp OR 'eye disease'/de OR 'cornea disease'/exp OR 'glaucoma'/exp OR 'intraocular pressure abnormality'/de OR 'optic nerve disease'/exp OR 'hemianopia'/exp OR 'visually impair*':ab,ti,kw OR 'visual impair*':ab,ti,kw OR blindness*:ab,ti,kw OR 'low vision*':ab,ti,kw OR 'reduced vision*':ab,ti,kw OR 'subnormal vision*':ab,ti,kw OR 'diminished vision*':ab,ti,kw OR 'visual disorder*':ab,ti,kw OR 'visually disab*':ab,ti,kw OR 'diabetic retinopath*':ab,ti,kw OR leber:ab,ti,kw OR 'leber s':ab,ti,kw OR lebers:ab,ti,kw OR 'retinal artery occlusion*':ab,ti,kw OR 'retinal degeneration*':ab,ti,kw OR 'retina degeneration*':ab,ti,kw OR 'macular degeneration*':ab,ti,kw OR 'macula degeneration*':ab,ti,kw OR 'macular dystroph*':ab,ti,kw OR 'macula dystroph*':ab,ti,kw OR maculopath*:ab,ti,kw OR 'macular edema*':ab,ti,kw OR 'macula edema*':ab,ti,kw OR 'macular oedema*':ab,ti,kw OR 'macula oedema*':ab,ti,kw OR 'retinitis pigmentosa':ab,ti,kw OR 'rod-cone dystroph*':ab,ti,kw OR 'cone-rod dystroph*':ab,ti,kw OR 'retinal detachment*':ab,ti,kw OR 'retina detachment*':ab,ti,kw OR 'retinal hemorrhage*':ab,ti,kw OR 'retinal haemorrhage*':ab,ti,kw OR 'retinal neovascularization*':ab,ti,kw OR 'retinal neovascularisation*':ab,ti,kw OR 'retinal vein occlusion*':ab,ti,kw OR vitreoretinopath*:ab,ti,kw OR 'vitreo-retinopath*':ab,ti,kw OR 'corneal disease*':ab,ti,kw OR 'cornea disease*':ab,ti,kw OR 'corneal disorder*':ab,ti,kw OR 'cornea disorder*':ab,ti,kw OR glaucoma*:ab,ti,kw OR 'optic nerve disease*':ab,ti,kw OR 'optic nerve disorder*':ab,ti,kw OR 'optic neuropath*':ab,ti,kw OR 'optic atroph*':ab,ti,kw OR ((vision NEAR/3 impair*):ab,ti,kw) OR ((vision NEAR/3 disorder*):ab,ti,kw) OR ((vision NEAR/3 disab*):ab,ti,kw) OR ((visual NEAR/3 disab*):ab,ti,kw) OR ((retina* NEAR/3 disease*):ab,ti,kw) OR ((retina* NEAR/3 disorder*):ab,ti,kw) OR hemianop*:ab,ti,kw OR quadrantanop*:ab,ti,kw OR adynamia:ab,ti,kw OR legasthenia:ab,ti,kw OR 'vision loss*':ab,ti,kw OR cataract*:ab,ti,kw | 647,059 |

**CINAHL (Ebsco) Session Results (27 Jan 2022)**

| Search | Query | Items found |
| --- | --- | --- |
| #5 | #4 NOT ((MH ("Adolescence" OR "Child+") OR TI (adolescen* OR child* OR schoolchild* OR infant* OR girl* OR boy* OR teen OR teens OR teenager* OR youth* OR pediatr* OR paediatr* OR puber*) OR AB (adolescen* OR child* OR schoolchild* OR infant* OR girl* OR boy* OR teen OR teens OR teenager* OR youth* OR pediatr* OR paediatr* OR puber*)) NOT (MH ("Adult+") OR TI (adult* OR man OR men OR woman OR women) OR AB (adult* OR man OR men OR woman OR women))) | 2,115 |
| #4 | #1 AND #2 AND #3 | 2,311 |
| #3 | (MH "Quality of Life+") OR (MH "Activities of Daily Living+") OR (MH "Self Care+") OR (MH "Health Status+") OR TI ("life qualit*" OR "quality of life" OR "activities of daily living" OR "daily living activit*" OR adl OR "chronic limitation of activity" OR "self care*" OR "health status" OR "level of health" OR "health level*" OR qol OR hrql OR hrqol OR participation OR handicap OR productivity OR reintegration OR "re-integration") OR AB ("life qualit*" OR "quality of life" OR "activities of daily living" OR "daily living activit*" OR adl OR "chronic limitation of activity" OR "self care*" OR "health status" OR "level of health" OR "health level*" OR qol OR hrql OR hrqol OR participation OR handicap OR productivity OR reintegration OR "re-integration") OR SU ("life qualit*" OR "quality of life" OR "activities of daily living" OR "daily living activit*" OR adl OR "chronic limitation of activity" OR "self care*" OR "health status" OR "level of health" OR "health level*" OR qol OR hrql OR hrqol OR participation OR handicap OR productivity OR reintegration OR "re-integration") | 582,544 |
| #2 | (MH "Disability Evaluation+") OR (MH "International Classification of Functioning, Disability, and Health") OR (MH "Surveys") OR (MH "Health Screening") OR (MH "Vision Screening") OR (MH "Questionnaires+") OR (MH "Scales") OR TI (questionnaire* OR "outcome measure*" OR scale* OR survey*) OR AB (questionnaire* OR "outcome measure*" OR scale* OR survey*) OR SU (questionnaire* OR "outcome measure*" OR scale* OR survey*) | 1,307,618 |
| #1 | (MH "Vision Disorders") OR (MH "Blindness+") OR (MH "Vision, Subnormal") OR (MH "Rehabilitation of Vision Impaired") OR (MH "Retinal Diseases") OR (MH "Retinal Artery Occlusion") OR (MH "Retinal Detachment") OR (MH "Cone-Rod Dystrophies") OR (MH "Diabetic Retinopathy") OR (MH "Leber's Congenital Amaurosis") OR (MH "Macular Degeneration") OR (MH "Retinal Vein Occlusion") OR (MH "Retinitis Pigmentosa") OR (MH "Eye Hemorrhage") OR (MH "Eye Diseases") OR (MH "Corneal Diseases+") OR (MH "Eye Diseases, Hereditary+") OR (MH "Ocular Hypertension+") OR (MH "Optic Nerve Diseases+") OR  TI ("visually impair*" OR "visual impair*" OR "vision impair*" OR blindness* OR "low vision*" OR "reduced vision*" OR "subnormal vision*" OR "diminished vision*" OR "vision disorder*" OR "visual disorder*" OR "vision disab*" OR "visual disab*" OR "visually disab*" OR "retinal disease*" OR "retina disease*" OR "retinal disorder*" OR "retina disorder*" OR "diabetic retinopath*" OR leber OR leber's OR lebers OR "retinal artery occlusion*" OR "retinal degeneration*" OR "retina degeneration*" OR "macular degeneration*" OR "macula degeneration*" OR "macular dystroph*" OR "macula dystroph*" OR maculopath* OR "macular edema*" OR "macula edema*" OR "macular oedema*" OR "macula oedema*" OR "retinitis pigmentosa" OR "Rod-Cone dystroph*" OR "Cone-Rod dystroph*" OR "retinal detachment*" OR "retina detachment*" OR "retinal hemorrhage*" OR "retinal haemorrhage*" OR "retinal neovascularization*" OR "retinal neovascularisation*" OR "retinal vein occlusion*" OR vitreoretinopath* OR "vitreo-retinopath*" OR "corneal disease*" OR "cornea disease*" OR "corneal disorder*" OR "cornea disorder*" OR glaucoma* OR "optic nerve disease*" OR "optic nerve disorder*" OR "optic neuropath*" OR "optic atroph*" OR hemianop* OR quadrantanop* OR adynamia OR legasthenia OR "vision loss*" OR cataract*) OR AB ("visually impair*" OR "visual impair*" OR "vision impair*" OR blindness* OR "low vision*" OR "reduced vision*" OR "subnormal vision*" OR "diminished vision*" OR "vision disorder*" OR "visual disorder*" OR "vision disab*" OR "visual disab*" OR "visually disab*" OR "retinal disease*" OR "retina disease*" OR "retinal disorder*" OR "retina disorder*" OR "diabetic retinopath*" OR leber OR leber's OR lebers OR "retinal artery occlusion*" OR "retinal degeneration*" OR "retina degeneration*" OR "macular degeneration*" OR "macula degeneration*" OR "macular dystroph*" OR "macula dystroph*" OR maculopath* OR "macular edema*" OR "macula edema*" OR "macular oedema*" OR "macula oedema*" OR "retinitis pigmentosa" OR "Rod-Cone dystroph*" OR "Cone-Rod dystroph*" OR "retinal detachment*" OR "retina detachment*" OR "retinal hemorrhage*" OR "retinal haemorrhage*" OR "retinal neovascularization*" OR "retinal neovascularisation*" OR "retinal vein occlusion*" OR vitreoretinopath* OR "vitreo-retinopath*" OR "corneal disease*" OR "cornea disease*" OR "corneal disorder*" OR "cornea disorder*" OR glaucoma* OR "optic nerve disease*" OR "optic nerve disorder*" OR "optic neuropath*" OR "optic atroph*" OR hemianop* OR quadrantanop* OR adynamia OR legasthenia OR "vision loss*" OR cataract*) OR SU ("visually impair*" OR "visual impair*" OR "vision impair*" OR blindness* OR "low vision*" OR "reduced vision*" OR "subnormal vision*" OR "diminished vision*" OR "vision disorder*" OR "visual disorder*" OR "vision disab*" OR "visual disab*" OR "visually disab*" OR "retinal disease*" OR "retina disease*" OR "retinal disorder*" OR "retina disorder*" OR "diabetic retinopath*" OR leber OR leber's OR lebers OR "retinal artery occlusion*" OR "retinal degeneration*" OR "retina degeneration*" OR "macular degeneration*" OR "macula degeneration*" OR "macular dystroph*" OR "macula dystroph*" OR maculopath* OR "macular edema*" OR "macula edema*" OR "macular oedema*" OR "macula oedema*" OR "retinitis pigmentosa" OR "Rod-Cone dystroph*" OR "Cone-Rod dystroph*" OR "retinal detachment*" OR "retina detachment*" OR "retinal hemorrhage*" OR "retinal haemorrhage*" OR "retinal neovascularization*" OR "retinal neovascularisation*" OR "retinal vein occlusion*" OR vitreoretinopath* OR "vitreo-retinopath*" OR "corneal disease*" OR "cornea disease*" OR "corneal disorder*" OR "cornea disorder*" OR glaucoma* OR "optic nerve disease*" OR "optic nerve disorder*" OR "optic neuropath*" OR "optic atroph*" OR hemianop* OR quadrantanop* OR adynamia OR legasthenia OR "vision loss*" OR cataract*) | 80,268 |

**APA PsycInfo (Ebsco) Session Results (27 Jan 2022)**

| Search | Query | Items found |
| --- | --- | --- |
| #5 | #4 NOT ((ZG ("adolescence (13-17 yrs)" OR "childhood (birth-12 yrs)" OR "infancy (2-23 mo)” OR "neonatal (birth-1 mo)" OR "preschool age (2-5 yrs)" OR "school age (6-12 yrs)") OR TI (adolescen* OR child* OR schoolchild* OR infant* OR girl* OR boy* OR teen OR teens OR teenager* OR youth* OR pediatr* OR paediatr* OR puber*) OR AB (adolescen* OR child* OR schoolchild* OR infant* OR girl* OR boy* OR teen OR teens OR teenager* OR youth* OR pediatr* OR paediatr* OR puber*)) NOT (ZG ("adulthood (18 yrs & older)" OR "aged (65 yrs & older)" OR "middle age (40-64 yrs)" OR "thirties (30-39 yrs)" OR "very old (85 yrs & older)") OR TI (adult* OR man OR men OR woman OR women) OR AB (adult* OR man OR men OR woman OR women))) | 657 |
| #4 | #1 AND #2 AND #3 | 747 |
| #3 | DE "Quality of Life" OR DE "Activities of Daily Living" OR DE "Daily Activities" OR DE "Self Care Skills" OR DE "Health Status" OR TI ("life qualit*" OR "quality of life" OR "activities of daily living" OR "daily living activit*" OR adl OR "chronic limitation of activity" OR "self care*" OR "health status" OR "level of health" OR "health level*" OR qol OR hrql OR hrqol OR participation OR handicap OR productivity OR reintegration OR "re-integration") OR AB ("life qualit*" OR "quality of life" OR "activities of daily living" OR "daily living activit*" OR adl OR "chronic limitation of activity" OR "self care*" OR "health status" OR "level of health" OR "health level*" OR qol OR hrql OR hrqol OR participation OR handicap OR productivity OR reintegration OR "re-integration") OR KW ("life qualit*" OR "quality of life" OR "activities of daily living" OR "daily living activit*" OR adl OR "chronic limitation of activity" OR "self care*" OR "health status" OR "level of health" OR "health level*" OR qol OR hrql OR hrqol OR participation OR handicap OR productivity OR reintegration OR "re-integration") | 280,300 |
| #2 | DE "Disability Evaluation" OR DE "International Classification of Diseases" OR DE "Surveys" OR DE "Questionnaires" OR TI (questionnaire* OR "outcome measure*" OR scale* OR survey*) OR AB (questionnaire* OR "outcome measure*" OR scale* OR survey*) OR KW (questionnaire* OR "outcome measure*" OR scale* OR survey*) | 910,444 |
| #1 | DE "Partially Sighted" OR DE "Vision Disorders" OR DE "Blind" OR DE "Eye Disorders" OR DE "Refraction Errors" OR DE "Glaucoma" OR DE "Optic Neuritis" OR DE "Hemianopia" OR TI ("visually impair*" OR "visual impair*" OR "vision impair*" OR blindness* OR "low vision*" OR "reduced vision*" OR "subnormal vision*" OR "diminished vision*" OR "vision disorder*" OR "visual disorder*" OR "vision disab*" OR "visual disab*" OR "visually disab*" OR "retinal disease*" OR "retina disease*" OR "retinal disorder*" OR "retina disorder*" OR "diabetic retinopath*" OR leber OR leber's OR lebers OR "retinal artery occlusion*" OR "retinal degeneration*" OR "retina degeneration*" OR "macular degeneration*" OR "macula degeneration*" OR "macular dystroph*" OR "macula dystroph*" OR maculopath* OR "macular edema*" OR "macula edema*" OR "macular oedema*" OR "macula oedema*" OR "retinitis pigmentosa" OR "Rod-Cone dystroph*" OR "Cone-Rod dystroph*" OR "retinal detachment*" OR "retina detachment*" OR "retinal hemorrhage*" OR "retinal haemorrhage*" OR "retinal neovascularization*" OR "retinal neovascularisation*" OR "retinal vein occlusion*" OR vitreoretinopath* OR "vitreo-retinopath*" OR "corneal disease*" OR "cornea disease*" OR "corneal disorder*" OR "cornea disorder*" OR glaucoma* OR "optic nerve disease*" OR "optic nerve disorder*" OR "optic neuropath*" OR "optic atroph*" OR hemianop* OR quadrantanop* OR adynamia OR legasthenia OR "vision loss*" OR cataract*) OR AB ("visually impair*" OR "visual impair*" OR "vision impair*" OR blindness* OR "low vision*" OR "reduced vision*" OR "subnormal vision*" OR "diminished vision*" OR "vision disorder*" OR "visual disorder*" OR "vision disab*" OR "visual disab*" OR "visually disab*" OR "retinal disease*" OR "retina disease*" OR "retinal disorder*" OR "retina disorder*" OR "diabetic retinopath*" OR leber OR leber's OR lebers OR "retinal artery occlusion*" OR "retinal degeneration*" OR "retina degeneration*" OR "macular degeneration*" OR "macula degeneration*" OR "macular dystroph*" OR "macula dystroph*" OR maculopath* OR "macular edema*" OR "macula edema*" OR "macular oedema*" OR "macula oedema*" OR "retinitis pigmentosa" OR "Rod-Cone dystroph*" OR "Cone-Rod dystroph*" OR "retinal detachment*" OR "retina detachment*" OR "retinal hemorrhage*" OR "retinal haemorrhage*" OR "retinal neovascularization*" OR "retinal neovascularisation*" OR "retinal vein occlusion*" OR vitreoretinopath* OR "vitreo-retinopath*" OR "corneal disease*" OR "cornea disease*" OR "corneal disorder*" OR "cornea disorder*" OR glaucoma* OR "optic nerve disease*" OR "optic nerve disorder*" OR "optic neuropath*" OR "optic atroph*" OR hemianop* OR quadrantanop* OR adynamia OR legasthenia OR "vision loss*" OR cataract*) OR KW ("visually impair*" OR "visual impair*" OR "vision impair*" OR blindness* OR "low vision*" OR "reduced vision*" OR "subnormal vision*" OR "diminished vision*" OR "vision disorder*" OR "visual disorder*" OR "vision disab*" OR "visual disab*" OR "visually disab*" OR "retinal disease*" OR "retina disease*" OR "retinal disorder*" OR "retina disorder*" OR "diabetic retinopath*" OR leber OR leber's OR lebers OR "retinal artery occlusion*" OR "retinal degeneration*" OR "retina degeneration*" OR "macular degeneration*" OR "macula degeneration*" OR "macular dystroph*" OR "macula dystroph*" OR maculopath* OR "macular edema*" OR "macula edema*" OR "macular oedema*" OR "macula oedema*" OR "retinitis pigmentosa" OR "Rod-Cone dystroph*" OR "Cone-Rod dystroph*" OR "retinal detachment*" OR "retina detachment*" OR "retinal hemorrhage*" OR "retinal haemorrhage*" OR "retinal neovascularization*" OR "retinal neovascularisation*" OR "retinal vein occlusion*" OR vitreoretinopath* OR "vitreo-retinopath*" OR "corneal disease*" OR "cornea disease*" OR "corneal disorder*" OR "cornea disorder*" OR glaucoma* OR "optic nerve disease*" OR "optic nerve disorder*" OR "optic neuropath*" OR "optic atroph*" OR hemianop* OR quadrantanop* OR adynamia OR legasthenia OR "vision loss*" OR cataract*) | 26,121 |

**Web of Science (Core Collection) Session Results (27 Jan 2022)**

| Search | Query | Items found |
| --- | --- | --- |
| #5 | #4 NOT TS=((adolescen* OR child* OR schoolchild* OR infant* OR girl* OR boy* OR teen OR teens OR teenager* OR youth* OR pediatr* OR paediatr* OR puber*) NOT (adult* OR man OR men OR woman OR women)) | 3,772 |
| #4 | #1 AND #2 AND #3 | 4,141 |
| #3 | TS=("life qualit*" OR "quality of life" OR "activities of daily living" OR "daily living activit*" OR adl OR "chronic limitation of activity" OR "self care*" OR "health status" OR "level of health" OR "health level*" OR qol OR hrql OR hrqol OR participation OR handicap OR productivity OR reintegration OR "re-integration") | 1,240,908 |
| #2 | TS=(questionnaire* OR "outcome measure*" OR scale* OR survey*) | 4,136,501 |
| #1 | TS=("visually impair*" OR "visual impair*" OR "vision impair*" OR blindness* OR "low vision*" OR "reduced vision*" OR "subnormal vision*" OR "diminished vision*" OR "vision disorder*" OR "visual disorder*" OR "vision disab*" OR "visual disab*" OR "visually disab*" OR "retinal disease*" OR "retina disease*" OR "retinal disorder*" OR "retina disorder*" OR "diabetic retinopath*" OR leber OR leber's OR lebers OR "retinal artery occlusion*" OR "retinal degeneration*" OR "retina degeneration*" OR "macular degeneration*" OR "macula degeneration*" OR "macular dystroph*" OR "macula dystroph*" OR maculopath* OR "macular edema*" OR "macula edema*" OR "macular oedema*" OR "macula oedema*" OR "retinitis pigmentosa" OR "Rod-Cone dystroph*" OR "Cone-Rod dystroph*" OR "retinal detachment*" OR "retina detachment*" OR "retinal hemorrhage*" OR "retinal haemorrhage*" OR "retinal neovascularization*" OR "retinal neovascularisation*" OR "retinal vein occlusion*" OR vitreoretinopath* OR "vitreo-retinopath*" OR "corneal disease*" OR "cornea disease*" OR "corneal disorder*" OR "cornea disorder*" OR glaucoma* OR "optic nerve disease*" OR "optic nerve disorder*" OR "optic neuropath*" OR "optic atroph*" OR hemianop* OR quadrantanop* OR adynamia OR legasthenia OR "vision loss*" OR cataract*) | 303,385 |

## Supplementary material: Appendix B: Included articles

Abateneh, A., Tesfaye, M., Bekele, S., & Gelaw, Y. (2013). Vision Loss and Psychological Distress among Ethiopians Adults: A Comparative Cross-Sectional Study. *PLoS ONE*, *8*(10), 1–7. https://doi.org/10.1371/journal.pone.0078335

Abd Rahman, M. H. (2020). Mental Health State of Low Vision Patients using the Hospital Anxiety and Depression Scale and The Depression, Anxiety and Stress Scale. *Medicine & Health*, *15*(1), 198–207. https://doi.org/10.17576/MH.2020.1501.18

Abraham, C. H., Boadi-Kusi, B., Morny, E. K. A., & Agyekum, P. (2021). Smartphone usage among people living with severe visual impairment and blindness. *Assistive Technology*. https://doi.org/10.1080/10400435.2021.1907485

Acton, J. H., Molik, B., Court, H., & Margrain, T. H. (2016). Effect of a home visit-based low vision rehabilitation intervention on visual function outcomes: An exploratory randomized controlled trial. *Investigative Ophthalmology and Visual Science*, *57*(15), 6662–6667. https://doi.org/10.1167/iovs.16-19901

Adam, I. (2018). Leisure aspirations of people with visual impairment in the Kumasi Metropolis, Ghana. *Annals of Leisure Research*, *21*(3), 347–363. https://doi.org/10.1080/11745398.2017.1387798

Adeyemo, O., Jeter, P. E., Rozanski, C., Arnold, E., Dalvin, L. A., Swenor, B., & Dagnelie, G. (2017). Living with Ultra-Low Vision: An Inventory of Self-Reported Visually Guided Activities by Individuals with Profound Visual Impairment. *Translational Vision Science & Technology*, *6*(3), 10. https://doi.org/10.1167/tvst.6.3.10

Adnan, T. H., Mohamed Apandi, M., Kamaruddin, H., Salowi, M. A., Law, K. B., Haniff, J., & Goh, P. P. (2018). Catquest-9SF questionnaire: validation of Malay and Chinese-language versions using Rasch analysis. *Health and Quality of Life Outcomes*, *16*(1), 5. https://doi.org/10.1186/s12955-017-0833-3

Akuffo, K. O., Sewpaul, R., Darrah, S., Dukhi, N., Kumah, D. ben, Agyei-Manu, E., Addo, E. K., Asare, A. K., Osei Duah, I., & Reddy, P. (2021). Vision loss, vision difficulty and psychological distress in South Africa: results from SANHANES-1. *BMC Psychology*, *9*(1). https://doi.org/10.1186/s40359-021-00558-x

Al-Dairi, W., al Sowayigh, O. M., al Saeed, A. A., & Alsaad, A. (2020). Depression Among Keratoconus Patients in Saudi Arabia. *Cureus*. https://doi.org/10.7759/cureus.11932

Alexis G. Malkin, Judith E. Goldstein, Monica Perlmutter, & Robert W. Massof. (2019). Responsiveness of the EQ-5D to the Effects of Low Vision Rehabilitation. *Optom Vis Sci.*, *90*(8), 799–805. https://doi.org/10.1097/OPX.0000000000000005.Responsiveness

Alma, M. A., van der Mei, S. F., Feitsma, W. N., Groothoff, J. W., van Tilburg, T. G., & Suurmeijer, T. P. B. M. (2011). Loneliness and self-management abilities in the visually impaired elderly. *Journal of Aging and Health*, *23*(5), 843–861. https://doi.org/10.1177/0898264311399758

Alma, M. A., van der Mei, S. F., Melis-Dankers, B. J. M., van Tilburg, T. G., Groothoff, J. W., & Suurmeijer, T. P. B. M. (2010). Participation of the elderly after vision loss. *Disability and Rehabilitation*, *33*(1), 63–72. https://doi.org/10.3109/09638288.2010.488711

Alshatrat, S., Bakri, I. al, Omari, W. al, & Tabnjh, A. (2021). Oral Health Knowledge, Behaviour, and Access to Dental Care in Visually Impaired Individuals in Jordan: A Case-Control Study. *The Open Dentistry Journal*, *15*(1), 33–40. https://doi.org/10.2174/1874210602115010033

Altangerel, U., Spaeth, G. L., & Rhee, D. J. (2003). Visual function, disability, and psychological impact of glaucoma. *Current Opinion in Ophthalmology*, *14*(2), 100–105. https://doi.org/10.1097/00055735-200304000-00009

Alworikat, N. A., Abduljaber, S. B., & Darawsheh, W. (2020). Quality of Life and Academic Experiences of Students with Visual Impairments Validation of the Arabic version of vocal tract discomfort scale Validation of the Arabic version of vocal tract discomfort scale View project Community-Based Rehabilitation (CBR) and Disability View project. *Article in Research Journal of Medical Sciences*. https://doi.org/10.36478/rjmsci.2020.77.83

Armbrecht, A. M., Aspinall, P. A., & Dhillon, B. (2004). A prospective study of visual function and quality of life following PDT in patients with wet age related macular degeneration. *British Journal of Ophthalmology*, *88*(10), 1270–1273. https://doi.org/10.1136/bjo.2003.038604

Arruda, J. S. D., Tibúrcio, J. D., de Campos-Carli, S. M., Teixeira, A. L., & Vasconcelos-Santos, D. V. (2021). Vision-Related Quality Of Life And Depression In Brazilian Patients With Toxoplasmic Retinochoroiditis: Ocular Toxoplasmosis, Quality Of Life And Depression. *International Journal of Infectious Diseases*, *112*, 66–72. https://doi.org/10.1016/j.ijid.2021.08.051

Au, C. P. Y., Fardell, N., Williams, M., Fraser-Bell, S., Campain, A., & Gillies, M. (2015). Patient experiences in retinal trials: A cross-sectional study. *BMC Ophthalmology*, *15*(1). https://doi.org/10.1186/s12886-015-0071-6

Awdeh, R. M., Elsing, S. H., Deramo, V. A., Stinnett, S., Lee, P. P., & Fekrat, S. (2010a). Vision-related quality of life in persons with unilateral branch retinal vein occlusion using the 25-item National Eye Institute Visual Function Questionnaire. *British Journal of Ophthalmology*, *94*(3), 319–323. https://doi.org/10.1136/bjo.2007.135913

Awdeh, R. M., Elsing, S. H., Deramo, V. A., Stinnett, S., Lee, P. P., & Fekrat, S. (2010b). Vision-related quality of life in persons with unilateral branch retinal vein occlusion using the 25-item National Eye Institute Visual Function Questionnaire. *British Journal of Ophthalmology*, *94*(3), 319–323. https://doi.org/10.1136/bjo.2007.135913

Ayaki, M., Tsubota, K., Kawashima, M., Kishimoto, T., Mimura, M., & Negishi, K. (2018). Sleep disorders are a prevalent and serious comorbidity in dry eye. *Investigative Ophthalmology and Visual Science*, *59*(14 Special Issue), DES143–DES150. https://doi.org/10.1167/iovs.17-23467

Ayele, F. A., Zeraye, B., Assefa, Y., Legesse, K., Azale, T., & Burton, M. J. (2017). The impact of glaucoma on quality of life in Ethiopia: A case-control study. *BMC Ophthalmology*, *17*(1). https://doi.org/10.1186/s12886-017-0643-8

Backman, H., & Williams, R. (2002). Living with Age-Related Macular Degeneration. *Journal of Visual Impairment and Blindness*, *96*(5), 345–348. https://doi.org/https://doi.org/10.1177/0145482X0209600506

Bahmani-Kashkouli, M., Pakdel, F., Astaraki, A., Hashemi, M., Honarbakhsh, Y., Mirarmandehi, B., & Jam, S. (2009). Quality of life in patients with thyroid eye disease. *Journal of Ophthalmic and Vision Research*, *4*(3), 164–168.

Baig, S., Diniz-Filho, A., Wu, Z., Abe, R. Y., Gracitelli, C. P. B., Cabezas, E., & Medeiros, F. A. (2016). Association of fast visual field loss with risk of falling in patients with glaucoma. *JAMA Ophthalmology*, *134*(8), 880–886. https://doi.org/10.1001/jamaophthalmol.2016.1659

Balarabe, A. H., Mahmoud, A. O., & Ayanniyi, A. A. (2014). The Sokoto blind beggars: Causes of blindness and barriers to rehabilitation services. *Middle East African Journal of Ophthalmology*, *21*(2), 147–152. https://doi.org/10.4103/0974-9233.129764

Ballemans, J., Zijlstra, G. A. R., van Rens, G. H. M. B., Schouten, J. S. A. G., & Kempen, G. I. J. M. (2012). Usefulness and acceptability of a standardised orientation and mobility training for partially-sighted older adults using an identification cane. *BMC Health Services Research*, *12*(1), 1. https://doi.org/10.1186/1472-6963-12-141

Bansback, N., Czoski-Murray, C., Carlton, J., Lewis, G., Hughes, L., Espallargues, M., Brand, C., & Brazier, J. (2007). Determinants of health related quality of life and health state utility in patients with age related macular degeneration: The association of contrast sensitivity and visual acuity. *Quality of Life Research*, *16*(3), 533–543. https://doi.org/10.1007/s11136-006-9126-8

Barber, C., Gould, C., Guillermo, G., Dupree, J., McLeer, M., Benevides, T., & Rosche, M. (2021). Interventions in the Scope of Occupational Therapy to Improve Psychosocial Well-Being in Older Adults with Low Vision and Mental Health Concerns: A Systematic Review. *Occupational Therapy in Health Care*, *35*(4), 397–423. https://doi.org/10.1080/07380577.2021.1946733

Bastawrous, A., Mathenge, W., Wing, K., Rono, H., Gichangi, M., Weiss, H. A., Macleod, D., Foster, A., Burton, M. J., & Kuper, H. (2016). Six-year incidence of blindness and visual impairment in Kenya: The Nakuru eye disease cohort study. *Investigative Ophthalmology and Visual Science*, *57*(14), 5974–5983. https://doi.org/10.1167/iovs.16-19835

Batra, A., Kain, R., Kumari, M., Paul, R., Dhawan, D., & Bakhshi, S. (2016). Parents’ Perspective of Quality of Life of Retinoblastoma Survivors. *Pediatric Blood and Cancer*, *63*(7), 1287–1289. https://doi.org/10.1002/pbc.25982

Bedard, K. M., Myrna, K. E., & Diehl, K. A. (2020). Preliminary evaluation of effect of two visual aid devices on navigation in blind dogs. *Journal of Small Animal Practice*, *61*(5), 308–315. https://doi.org/10.1111/jsap.13120

Berdeaux, G. H., Nordmann, J. P., Colin, E., & Arnould, B. (2005). Vision-related quality of life in patients suffering from age-related macular degeneration. *American Journal of Ophthalmology*, *139*(2), 271–279. https://doi.org/10.1016/j.ajo.2004.09.028

Berger, S. (2013). Effectiveness of occupational therapy interventions for older adults living with low vision. *American Journal of Occupational Therapy*, *67*(3), 263–265. https://doi.org/10.5014/ajot.2013.007203

Berger, S., & Porell, F. (2008). The association between low vision and function. *Journal of Aging and Health*, *20*(5), 504–525. https://doi.org/10.1177/0898264308317534

Berry, S., Mangione, C. M., Lindblad, A. S., & McDonnell, P. J. (2003). Development of the National Eye Institute Refractive Error Correction Quality of Life Questionnaire: Focus Groups. *Ophthalmology*, *110*(12), 2285–2291. https://doi.org/10.1016/j.ophtha.2003.08.021

Biggerstaff, K. S., & Lin, A. (2018). Glaucoma and Quality of Life. *International Ophthalmology Clinics*, *58*(3), 11–22. https://doi.org/10.1097/IIO.0000000000000230

Black, A. A., Wood, J. M., & Lovie-Kitchin, J. E. (2011). Inferior visual field reductions are associated with poorer functional status among older adults with glaucoma. *Ophthalmic and Physiological Optics*, *31*(3), 283–291. https://doi.org/10.1111/j.1475-1313.2010.00811.x

Blair, E., & Smithers-Sheedy, H. (2016). Strabismus, a preventable barrier to social participation: A short report. *Developmental Medicine and Child Neurology*, *58*, 57–59. https://doi.org/10.1111/dmcn.13020

Blondeau, P., Esper, P., & Mazerolle, É. (2007). An information session for glaucoma patients. *Canadian Journal of Ophthalmology*, *42*(6), 816–820. https://doi.org/10.3129/I07-154

Boadi-Kusi, S. B., Hansraj, R., Kumi-Kyereme, A., Mashige, K. P., Awusabo-Asare, K., Ocansey, S., & Kyei, S. (2014). Ocular Health Assessment of Cocoa Farmers in a Rural Community in Ghana. *Journal of Agromedicine*, *19*(2), 171–180. https://doi.org/10.1080/1059924X.2014.886537

Boerner, K., & Reinhardt, J. P. (2003). Giving while in need: Support provided by disabled older adults. *Journals of Gerontology - Series B Psychological Sciences and Social Sciences*, *58*(5), 297–304. https://doi.org/10.1093/geronb/58.5.S297

Boey, D., Tse, T., Lim, Y. hui, Chan, M. L., Fitzmaurice, K., & Carey, L. (2021). The impact of low vision on activities, participation, and goals among older adults: a scoping review. In *Disability and Rehabilitation*. Taylor and Francis Ltd. https://doi.org/10.1080/09638288.2021.1937340

Bonnielin K. Swenor, Karen Bandeen-Roche, Beatriz Muñoz, & Sheila K. West. (2014). Does Walking Speed Mediate The Association Between VisualImpairment and Self-Report of Mobility Disability? The SalisburyEye Evaluation Study. *J Am Geriatr Soc*, *62*(8), 1540–1545. https://doi.org/10.1111/jgs.12937.Does

Bookwala, J. (2011). Marital quality as a moderator of the effects of poor vision on quality of life among older adults. *Journals of Gerontology - Series B Psychological Sciences and Social Sciences*, *66 B*(5), 605–616. https://doi.org/10.1093/geronb/gbr091

Borkenstein, A. F., Borkenstein, E. M., Persson, S., Muus, G., & Nielsen, N. v. (2021). Improving outcomes for patients with age-related macular degeneration and cataracts: The importance of including an assessment of activities of daily life (adl). In *Clinical Ophthalmology*, 15, 3333–3339. https://doi.org/10.2147/OPTH.S327274

Bourne, R. R. A., Flaxman, S. R., Braithwaite, T., Cicinelli, M. v., Das, A., Jonas, J. B., Keeffe, J., Kempen, J., Leasher, J., Limburg, H., Naidoo, K., Pesudovs, K., Resnikoff, S., Silvester, A., Stevens, G. A., Tahhan, N., Wong, T., Taylor, H. R., Ackland, P., … Zheng, Y. (2017). Magnitude, temporal trends, and projections of the global prevalence of blindness and distance and near vision impairment: a systematic review and meta-analysis. *The Lancet Global Health*, *5*(9), e888–e897. https://doi.org/10.1016/S2214-109X(17)30293-0

Bradley E. Dougherty, S. R. M. C. B. K. L. A. J. T. W. R. M. A. B. Mco. (2009). The Development of a Battery of Functional Tests for Low Vision. *Optom Vis Sci*, *86*(8), 955–963. https://doi.org/10.1097/OPX.0b013e3181b180a6

Braich, P. S., Lal, V., Hollands, S., & Almeida, D. R. (2012). Burden and depression in the caregivers of blind patients in India. *Ophthalmology*, *119*(2), 221–226. https://doi.org/10.1016/j.ophtha.2011.07.038

Braithwaite, T., Wiegerinck, Np. A., Denni, & ston, A. (2020). Vision Loss from Atypical Optic Neuritis: Patient and Physician Perspectives. *Opthalmol Ther*, *9*(2), 215–220. https://doi.org/10.1007/s40123-020-00247-9

Brandão, G. S., Oliveira, L. V. F., Brandão, G. S., Silva, A. S., Sampaio, A. A. C., Urbano, J. J., Soares, A., Santos Faria, N., Pasqualotto, L. T., Oliveira, E. F., Oliveira, R. F., Pires-Oliveira, D. A. A., & Camelier, A. A. (2018). Effect of a home-based exercise program on functional mobility and quality of life in elderly people: Protocol of a single-blind, randomized controlled trial. *Trials*, *19*(1). https://doi.org/10.1186/s13063-018-3061-1

Brennan, M. (2002). Spirituality and psychosocial development in middle-age and older adults with vision loss. *Journal of Adult Development*, *9*(1), 31–46. https://doi.org/10.1023/A:1013825217305

Broman, A. T., Munoz, B., Rodriguez, J., Sanchez, R., Quigley, H. A., Klein, R., Snyder, R., & West, S. K. (2002). The impact of visual impairment and eye disease on vision-related quality of life in a Mexican-American population: Proyecto VER. *Investigative Ophthalmology and Visual Science*, *43*(11), 3393–3398.

Browne, C., Brazier, J., Carlton, J., Alavi, Y., & Jofre-Bonet, M. (2012). Estimating quality-adjusted life years from patient-reported visual functioning. *Eye (Basingstoke)*, *26*(10), 1295–1301. https://doi.org/10.1038/eye.2012.137

Brown, M. M., Brown, G. C., Sharma, S., & Busbee, B. (2003). Quality of life associated with visual loss: A time tradeoff utility analysis comparison with medical health states. *Ophthalmology*, *110*(6), 1076–1081. https://doi.org/10.1016/S0161-6420(03)00254-9

Brown, M. M., Brown, G. C., Sharma, S., Landy, J., & Bakal, J. (2002). Quality of life with visual acuity loss from diabetic retinopathy and age-related macular degeneration. *Archives of Ophthalmology*, *120*(4), 481–484. https://doi.org/10.1001/archopht.120.4.481

Bruijning, Janna E. Ger van rens, Dirk Knol, and R. van N. (2013). Psychometric Analyses to Improve the Dutch ICF Activity Inventory. *Optometry and Vision Science*, *90*(8), 806–819.

Bruijning Janna, van Nispen Ruth, Knol Dirk, & van Rens Ger. (2012). Low_Vision_Rehabilitation_Plans_Comparing_Two.13. *OPTOMETRY AND VISION SCIENCE*, *89*(2), 203–2014.

Bruijning, J. E. (2014). Evaluation of reading, writing and watching TV using the dutch ICF activity inventory. *Optometry and Vision Science*, *91*(11), 1360–1371.

Bruijning, J. E., van Nispen, R. M., & van Rens, G. H. (2010). Feasibility of the Dutch ICF Activity Inventory: A pilot study. *BMC Health Services Research*, *10*(1), 318. https://doi.org/10.1186/1472-6963-10-318

Bruijning, J. E., van Rens, G. H. M. B., Fick, M., Knol, D. L., & van Nispen, R. M. A. (2014). Longitudinal observation, evaluation and interpretation of coping with mental (emotional) health in low vision rehabilitation using the Dutch ICF Activity Inventory. *Health and Quality of Life Outcomes*, *12*(1), 1–16. https://doi.org/10.1186/s12955-014-0182-4

Bruijning, J., van Nispen, R., Verstraten, P., & van Rens, G. (2010). A Dutch ICF version of the activity inventory: Results from focus groups with visually impaired persons and experts. *Ophthalmic Epidemiology*, *17*(6), 366–377. https://doi.org/10.3109/09286586.2010.528133

Burggraaff, M. C., van Nispen, R. M. A., Hoeben, F. P., Knol, D. L., & van Rens, G. H. M. B. (2012). Randomized controlled trial on the effects of training in the use of closed-circuit television on reading performance. *Investigative Ophthalmology & Visual Science*, *53*(4), 2142–2150. https://doi.org/10.1167/iovs.11-8407

Burstedt, M. S. I., Monestam, E., & Sandgren, O. (2005). Associations between specific measures of vision and vision-related quality of life in patients with bothnia dystrophy, a defined type of retinitis pigmentosa. *Retina*, *25*(3), 317–323. https://doi.org/10.1097/00006982-200504000-00011

Burton, A. E., Gibson, J. M., & Shaw, R. L. (2016). How do older people with sight loss manage their general health? A qualitative study. *Disability and Rehabilitation*, *38*(23), 2277–2285. https://doi.org/10.3109/09638288.2015.1123310

Cahill, M. T., Banks, A. D., Stinnett, S. S., & Toth, C. A. (2005). Vision-related quality of life in patients with bilateral severe age-related macular degeneration. *Ophthalmology*, *112*(1), 152–158. https://doi.org/10.1016/j.ophtha.2004.06.036

Cai, C. X., Li, Y., Zeger, S. L., & McCarthy, M. L. (2021). Social determinants of health impacting adherence to diabetic retinopathy examinations. *BMJ Open Diabetes Research and Care*, *9*(1). https://doi.org/10.1136/bmjdrc-2021-002374

Çankaya, C. (2021). Examination of satisfaction levels of visually impaired athletes. *Revista on Line de Política e Gestão Educacional*, 1513–1523. https://doi.org/10.22633/rpge.v25i2.15499

Cankurtaran, V., Ozates, S., Ezerbolat Ozates, M., & Ozler, S. (2020). Influence of visual acuity level on sexual function in patients with cataract. *Indian Journal of Ophthalmology*, *68*(8), 1579–1583. https://doi.org/10.4103/ijo.IJO_2290_19

Chakrabarti S. (2018). Psychosocial aspects of colour vision deficiency: Implications for a career in medicine. *The National Medical Journal of India*, *31*(2), 86–96. http://www.nmji.in

Chan, E. W., Chiang, P. P. C., Wong, T. Y., Saw, S. M., Loon, S. C., Aung, T., & Lamoureux, E. (2013). Impact of glaucoma severity and laterality on vision-specific functioning: The Singapore Malay Eye Study. *Investigative Ophthalmology and Visual Science*, *54*(2), 1169–1175. https://doi.org/10.1167/iovs.12-10258

Chang, K. yi J., Dillon, L. L., Deverell, L., Boon, M. Y., & Keay, L. (2019). Orientation and mobility outcome measures. *Clinical and Experimental Optometry*, *103*(4), 434–448. https://doi.org/10.1111/cxo.13004

Chang, M. Y., Velez, F. G., Demer, J. L., Isenberg, S. J., Coleman, A. L., & Pineles, S. L. (2015). Quality of life in adults with strabismus. *American Journal of Ophthalmology*, *159*(3), 539-544.e2. https://doi.org/10.1016/j.ajo.2014.12.003

Chan, T. L., Perlmutter, M. S., Andrews, M., Sunness, J. S., Goldstein, J. E., & Massof, R. W. (2015). Equating Visual Function Scales to Facilitate Reporting of Medicare Functional G-Code Severity/Complexity Modifiers for Low-Vision Patients. *Archives of Physical Medicine and Rehabilitation*, *96*(10), 1859–1865. https://doi.org/10.1016/j.apmr.2015.06.013

Chan, V. F., Naidoo, J., Chinanayi, F. S., & Naidoo, K. S. (2017). Near vision correction and quality of life among textile factory workers in Durban. *African Vision and Eye Health*, *76*(1). https://doi.org/10.4102/aveh.v76i1.384

Chen, E., Looman, M., Laouri, M., Gallagher, M., van Nuys, K., Lakdawalla, D., & Fortuny, J. (2010). Burden of illness of diabetic macular edema: Literature review. *Current Medical Research and Opinion*, *26*(7), 1587–1597. https://doi.org/10.1185/03007995.2010.482503

Chen, J., Lin, Z. N., Tao, Y. T., Zhao, Q. N., Li, Q., Yang, H., Xu, P., Chen, J. M., Ma, X. Q., & Cui, H. P. (2019). Influences of personality characteristics and coping modes on anxiety in primary glaucoma patients. *International Journal of Ophthalmology*, *12*(7), 1163–1169. https://doi.org/10.18240/ijo.2019.07.18

Chen, Y., Nondahl, D. M., Schubert, C. R., Klein, B. E. K., Klein, R., & Cruickshanks, K. J. (2017). The Relation between Sleep Disruption and Cataract in a Large Population-Based Study. *Ophthalmic Epidemiology*, *24*(2), 111–115. https://doi.org/10.1080/09286586.2016.1259640

Cheung, C. M. G., Li, X., Mathur, R., Lee, S. Y., Chan, C. M., Yeo, I., Loh, B. K., Williams, R., Wong, E. Y. M., Wong, D., & Wong, T. Y. (2014). A prospective study of treatment patterns and 1-year outcome of asian age-related macular degeneration and polypoidal choroidal vasculopathy. *PLoS ONE*, *9*(6). https://doi.org/10.1371/journal.pone.0101057

Chia, E. M., Wang, J. J., Rochtchina, E., Smith, W., Cumming, R. R., & Mitchell, P. (2004). Impact of Bilateral Visual Impairment on Health-Related Quality of Life: The Blue Mountains Eye Study. *Investigative Ophthalmology and Visual Science*, *45*(1), 71–76. https://doi.org/10.1167/iovs.03-0661

Choi, N. G., DiNitto, D. M., Lee, O. E. K., & Choi, B. Y. (2020). Internet and health information technology use and psychological distress among older adults with self-reported vision impairment: Case-control study. *Journal of Medical Internet Research*, *22*(6). https://doi.org/10.2196/17294

Choudhury, F., Varma, R., Klein, R., James Gauderman, W., Azen, S. P., McKean-Cowdin, R., Paz, S. H., Cisneros, L., Corona, E., Cuestas, C., Globe, D. R., Hahn, S., Lai, M. Y., Martinez, G., Preston-Martin, S., Smith, R. E., Tetrow, L., Torres, M., Uribe, N., … Meuer, S. M. (2016). Age-related macular degeneration and quality of life in latinos: The Los Angeles Latino Eye study. *JAMA Ophthalmology*, *134*(6), 683–690. https://doi.org/10.1001/jamaophthalmol.2016.0794

Chun, R., Bhakhri, R., Coalter, J., & Jay, W. M. (2012). Smartphone usage in patients with optic atrophy. *Neuro-Ophthalmology*, *36*(5), 193–195. https://doi.org/10.3109/01658107.2012.710923

Cieza, A., Geyh, S., Chatterji, S., Kostanjsek, N., Üstün, B., & Stucki, G. (2005). ICF linking rules: An update based on lessons learned. *Journal of Rehabilitation Medicine*, *37*(4), 212–218. https://doi.org/10.1080/16501970510040263

Cimarolli, V. R., & Wang, S. W. (2006). Differences in social support among employed and unemployed adults who are visually impaired. *Journal of Visual Impairment and Blindness*, *100*(9), 545–556. https://doi.org/10.1177/0145482x0610000906

Cingu, A. K., Bez, Y., Cinar, Y., Turkcu, F. M., Yildirim, A., Sahin, A., Tas, C., & Sir, A. (2015). Impact of collagen cross-linking on psychological distress and vision and health-related quality of life in patients with keratoconus. *Eye and Contact Lens*, *41*(6), 349–353. https://doi.org/10.1097/ICL.0000000000000129

Clayton, J. A., Eydelman, M., Vitale, S., Manukyan, Z., Kramm, R., Datiles, M., Temple, A., Murphy, E., Kim, J., Hilmantel, G., Rorer, E., Hammel, K., & Ferris, F. (2013). Web-based versus paper administration of common ophthalmic questionnaires: Comparison of subscale scores. *Ophthalmology*, *120*(10), 2151–2159. https://doi.org/10.1016/j.ophtha.2013.03.019

Clemons, T. E., Chew, E. Y., Bressler, S. B., McBee, W., & Sharma, S. (2003). National Eye Institute visual function questionnaire in the age-related eye disease study (AREDS). *Evidence-Based Eye Care*, *4*(3), 174–175. https://doi.org/10.1097/00132578-200307000-00025

Cmar, J. L., McDonnall, M. C., & Crudden, A. (2018). Transportation self-efficacy and employment among individuals with visual impairments. *Journal of Vocational Rehabilitation*, *48*(2), 257–268. https://doi.org/10.3233/JVR-180925

Cockerham, K. P., Padnick-Silver, L., Stuertz, N., Francis-Sedlak, M., & Holt, R. J. (2021). Quality of Life in Patients with Chronic Thyroid Eye Disease in the United States. *Ophthalmology and Therapy*, *10*(4), 975–987. https://doi.org/10.1007/s40123-021-00385-8

Coco-Martín, M. B., López-Miguel, A., Cuadrado, R., Mayo-Iscar, A., Herrero, A. J., Pastor, J. C., & Maldonado, M. J. (2017). Reading Performance Improvements in Patients with Central Vision Loss without Age-Related Macular Degeneration after Undergoing Personalized Rehabilitation Training. *Current Eye Research*, *42*(9), 1260–1268. https://doi.org/10.1080/02713683.2017.1315140

Colenbrander, A. (2010). Assessment of functional vision and its rehabilitation: Review Article. *Acta Ophthalmologica*, *88*(2), 163–173. https://doi.org/10.1111/j.1755-3768.2009.01670.x

Colgan, J. C., Bopp, M. J., Starkoff, B. E., & Lieberman, L. J. (2016). Fitness Wearables and Youths with Visual Impairments: Implications for Practice and Application. *Journal of Visual Impairment & Blindness*, *110*(5), 335–348.

Coney, J. M. (2019). Addressing Unmet Needs  in Diabetic Retinopathy. *The American Journal of Managed Care*, *25*(16), S311–S316. www.ajmc.com

Cordes, C., Heutink, J., Brookhuis, K. A., Brouwer, W. H., & Melis-Dankers, B. J. M. (2018). Mobility scooter driving ability in visually impaired individuals. *Disability and Rehabilitation*, *40*(12), 1372–1378. https://doi.org/10.1080/09638288.2017.1295471

Corn, A. L., & Rosenblum, L. P. (2002). Experiences of Older Adults who stopped driving beacuase of their Visual impairment. *Journal of Visual Impairment & Blindness*, *96*(7), 701–710. https://doi.org/https://doi.org/10.1177/0145482X0209600702

Corn L.A., & Rosenblum L.P. (2002). Experiences of Older Adults Who Stopped Driving Because of Their Visual Impairments: Part 2. *Journal of Visual Impairment & Blindnesss*, *96*(7), 485–500. https://doi.org/https://doi.org/10.1177/0145482X1611000505

Cortina, M. S., & Hallak, J. A. (2014). *Vision-Related Quality-of-Life Assessment Using NEI VFQ-25 in Patients After Boston Keratoprosthesis Implantation*. www.corneajrnl.com

Cox, T. M., & Ffytche, D. H. (2014). Negative outcome Charles Bonnet syndrome. *British Journal of Ophthalmology*, *98*(9), 1236–1239. https://doi.org/10.1136/bjophthalmol-2014-304920

Crabb, D. P. (2016). A view on glaucoma - Are we seeing it clearly? *Eye (Basingstoke)*, *30*(2), 304–313. https://doi.org/10.1038/eye.2015.244

Crews, J. E., & Campbell, V. A. (2001). Health conditions, activity limitations, and participation restrictions among older people with visual impairments. *Journal of Visual Impairment and Blindness*, *95*(8), 453–467. https://doi.org/10.1177/0145482x0109500802

Croft, E. (2020). Experiences of visually impaired and blind students in uk higher education: An exploration of access and participation. *Scandinavian Journal of Disability Research*, *22*(1), 382–392. https://doi.org/10.16993/SJDR.721

Crudden, A., Cmar, J. L., & Mcdonnall, M. C. (n.d.). *Stress Associated with Transportation: A Survey of Persons with Visual Impairments*. http://jvib.org/CEs.

Cruice, M., Worrall, L., & Hickson, L. (2005). Personal factors, communication and vision predict social participation in older adults. *International Journal of Speech-Language Pathology*, *7*(4), 220–232. https://doi.org/10.1080/14417040500337088

Cumurcu, T., Cumurcu, B. E., Celikel, F. C., & Etikan, I. (2006). Depression and anxiety in patients with pseudoexfoliative glaucoma. *General Hospital Psychiatry*, *28*(6), 509–515. https://doi.org/10.1016/j.genhosppsych.2006.09.004

Cypel, M. C., Kasahara, N., Atique, D., Umbelino, C. C., Alcântara, M. P. A., Seixas, F. S., Almeida, G. v., Mandia, C., & Cohen, R. (2004). Quality of life in patients with glaucoma who live in a developing country. *International Ophthalmology*, *25*(5–6), 267–272. https://doi.org/10.1007/s10792-005-0077-9

Dahlin-Ivanoff, S., Sonn, U., & Svensson, E. (2001). Development of an ADL instrument targeting elderly persons with age-related macular degeneration. *Disability and Rehabilitation*, *23*(2), 69–79. https://doi.org/10.1080/096382801750058152

Das, A., Quartilho, A., Xing, W., Bunce, C., Rubin, G., MacKenzie, K., Adams, G., Dahlmann-Noor, A., & Theodorou, M. (2018). Visual functioning in adults with Idiopathic Infantile Nystagmus Syndrome (IINS). *Strabismus*, *26*(4), 203–209. https://doi.org/10.1080/09273972.2018.1526958

Decarlo, D. K., Scilley, K., Wells, J., & Owsley, C. (2003). Driving habits and health-related quality of life in patients with age-related maculopathy. *Optometry and Vision Science*, *80*(3), 207–213. https://doi.org/10.1097/00006324-200303000-00010

Denoyer, A., Rabut, G., & Baudouin, C. (2012). Tear film aberration dynamics and vision-related quality of life in patients with dry eye disease. *Ophthalmology*, *119*(9), 1811–1818. https://doi.org/10.1016/j.ophtha.2012.03.004

Devenney, R., & O’Neill, S. (2011). The experience of diabetic retinopathy: A qualitative study. *British Journal of Health Psychology*, *16*(4), 707–721. https://doi.org/10.1111/j.2044-8287.2010.02008.x

Donovan, T. (2001). Is Having the Luck of Growing Old in the Gay, Lesbian, Bisexual, Transgender Community Good or Bad Luck? *Journal of Gay and Lesbian Social Services*, *13*(4), 19–22. https://doi.org/10.1300/J041v13n04

Douglas, G., Corcoran, C., & Pavey, S. (2007). The role of the WHO ICF as a framework to interpret barriers and to inclusion: Visually impaired people’s views and experiences of personal computers. *The British Journal of Visual Impairment*, *25*(1), 32–50. https://doi.org/10.1177/0264619607071773

Douglas, G., Pavey, S., Corcoran, C., & Clements, B. (2012). Evaluating the use of the ICF as a framework for interviewing people with a visual impairment about their mobility and travel. *The British Journal of Visual Impairment*, *30*(1), 6–21. https://doi.org/10.1177/0264619611428932

Draper, E. M., Feng, R., Appel, S. D., Graboyes, M., Engle, E., Ciner, E. B., Ellenberg, J. H., & || D. S. (n.d.). *Low Vision Rehabilitation for Adult African Americans in Two Settings*. http://journals.lww.com/optvissci

Dunbar, H. M., Crossland, M. D., Bunce, C., Egan, C., & Rubin, G. S. (2012). The effect of low vision rehabilitation in diabetic eye disease: A randomised controlled trial protocol. *Ophthalmic and Physiological Optics*, *32*(4), 282–293. https://doi.org/10.1111/j.1475-1313.2012.00914.x

Ekpenyong, B. N., Echendu, D., & Ekanem, E. (2020). Visual health status and its relationship with road traffic accidents amongst Nigerian vehicle drivers: A publication of the Nigerian Optometric Association. *African Vision and Eye Health*, *79*(1), 1–8. https://doi.org/10.4102/AVEH.V79I1.577

Elliott, A. F., Mcgwin, G., Kline, L. B., & Owsley, C. (2015). Vision impairment among older adults residing in subsidized housing communities. *Gerontologist*, *55*, S108–S117. https://doi.org/10.1093/geront/gnv028

Elliott, A. F., McGwin, G., Kline, L. B., & Owsley, C. (2015). Vision impairment among older adults residing in subsidized housing communities. *Gerontologist*, *55*, S108–S117. https://doi.org/10.1093/geront/gnv028

Elliott, D. B., Pesudovs, K., & Mallinson, T. (2007). Vision-Related Quality of Life. *Optometry and Vision Science*, *84*(8), 656–658.

Elsman, E. B. M., van Rens, G. H. M. B., & van Nispen, R. M. A. (2018). Psychometric properties of a new intake questionnaire for visually impaired young adults: The Participation and Activity Inventory for Young Adults (PAI-YA). *PLoS ONE*, *13*(8), 1–24. https://doi.org/10.1371/journal.pone.0201701

Elsman, E. B. M., van Rens, G. H. M. B., & van Nispen, R. M. A. (2019). Quality of life and participation of young adults with a visual impairment aged 18–25 years: comparison with population norms. *Acta Ophthalmologica*, *97*(2), 165–172. https://doi.org/10.1111/aos.13903

Enoch, J., Jones, L., Taylor, D. J., Bronze, C., Kirwan, J. F., Jones, P. R., & Crabb, D. P. (2020). How do different lighting conditions affect the vision and quality of life of people with glaucoma? A systematic review. *Eye*, 34(1), 138–154. https://doi.org/10.1038/s41433-019-0679-5

Eser-Öztürk, H., Yeter, V., Karabekiroğlu, A., & Süllü, Y. (2021). The Effect of Vision-Related Quality of Life on Depression and Anxiety in Patients with Behçet Uveitis. *Turkish Journal of Ophthalmology*, *51*(6), 358–364. https://doi.org/10.4274/tjo.galenos.2020.06791

Eszes, D. J., Szabó, D. J., Russell, G., Kirby, P., Paulik, E., Nagymajtényi, L., Facskó, A., Moe, M. C., & Petrovski, B. (2016). Diabetic Retinopathy Screening Using Telemedicine Tools: Pilot Study in Hungary. *Journal of Diabetes Research*, *2016*. https://doi.org/10.1155/2016/4529824

Evans, J. R., Fletcher, A. E., & Wormald, R. P. L. (2007). Depression and Anxiety in Visually Impaired Older People. *Ophthalmology*, *114*(2), 283–288. https://doi.org/10.1016/j.ophtha.2006.10.006

Falahaty, K., Cheong, L. S., & Mohd Isa, M. B. H. (2015). Disability among elderly people with visual impairment in two welfare homes in Malaysia. *Biomedical and Pharmacology Journal*, *8*(2), 1369–1382. https://doi.org/10.13005/bpj/897

Fayers, T., Abdullah, W., Walton, V., & Wilkins, M. R. (2009). Impact of written and photographic instruction sheets on patient behavior after cataract surgery. *Journal of Cataract and Refractive Surgery*, *35*(10), 1739–1743. https://doi.org/10.1016/j.jcrs.2009.07.003

Fenwick, E. K., Man, R. E. K., Cheung, C. M. G., Sabanayagam, C., Cheng, C. Y., Neelam, K., Chua, J., Gan, A. T. L., Mitchell, P., Wong, T. Y., & Lamoureux, E. L. (2017). Ethnic differences in the association between age-related macular degeneration and vision-specific functioning. *JAMA Ophthalmology*, *135*(5), 469–476. https://doi.org/10.1001/jamaophthalmol.2017.0266

Finger, R. P., Ayton, L. N., Deverell, L., McSweeney, S. C., Luu, C. D., Fenwick, E. K., Keeffe, J. E., Guymer, R. H., Bentley, S. A., & Dip, G. O. (2016). Developing a Very Low Vision Orientation and Mobility Test Battery (O&M-VLV). *Optometry and Vision Science*, *93*(9). www.optvissci.com

Finger, R. P., Fenwick, E., Owsley, C., Holz, F. G., & Lamoureux, E. L. (2011). Visual functioning and quality of life under low luminance: Evaluation of the german low luminance questionnaire. *Investigative Ophthalmology and Visual Science*, *52*(11), 8241–8249. https://doi.org/10.1167/iovs.11-7858

Finger, R. P., Fenwick, E., Pesudovs, K., Marella, M., Lamoureux, E. L., & Holz, F. G. (2012). Rasch analysis reveals problems with multiplicative scoring in the macular disease quality of life questionnaire. *Ophthalmology*, *119*(11), 2351–2357. https://doi.org/10.1016/j.ophtha.2012.05.031

Fontenot, J. L., Bona, M. D., Kaleem, M. A., McLaughlin, W. M., Morse, A. R., Schwartz, T. L., Shepherd, J. D., & Jackson, M. lou. (2018). Vision Rehabilitation Preferred Practice Pattern®. *Ophthalmology*, *125*(1), P228–P278. https://doi.org/10.1016/j.ophtha.2017.09.030

Forooghian Farzin, Agrón Elvira, Clemons Traci E., Ferris Frederick L., & Chew Emily Y. (2009). Visual Acuity Outcomes after Cataract Surgery in Patients withAge-Related Macular Degeneration: Age-Related Eye Disease. *Opthalmology*, *116*(11), 2093–2100. https://doi.org/10.1016/j.ophtha.2009.04.033

Fox, M. H., Krahn, G. L., Sinclair, L. B., & Cahill, A. (2015). How to use the ICF: A Practical Manual for using theInternational Classification of Functioning, Disability and Health(ICF). *Disability and Health Journal*, *8*(3), 457–463. https://doi.org/10.1016/j.dhjo.2015.03.002

Frennesson, C., Nilsson, U. L., Peebo, B. B., & Nilsson, S. E. G. (2010). Significant improvements in near vision, reading speed, central visual field and related quality of life after ranibizumab treatment of wet age-related macular degeneration. *Acta Ophthalmologica*, *88*(4), 420–425. https://doi.org/10.1111/j.1755-3768.2009.01576.x

Frost, N. A., Sparrow, J. M., Hopper, C. D., & Peters, T. J. (2001). Reliability of the VCM1 questionnaire when administered by post and by telephone. *Ophthalmic Epidemiology*, *8*(1), 1–11. https://doi.org/10.1076/opep.8.1.1.1539

Fujita, K., Suzukamo, Y., Murotani, K., Jinno, A., & Kamei, M. (2021). Impact of low luminance conditions on quality of life for the visually impaired: development of the Low Luminance Questionnaire Japanese version. *Japanese Journal of Ophthalmology*, *65*(4), 554–560. https://doi.org/10.1007/s10384-021-00838-4

Gabrielian, A., Hariprasad, S. M., Jager, R. D., Green, J. L., & Mieler, W. F. (2010). The utility of visual function questionnaire in the assessment of the impact of diabetic retinopathy on vision-related quality of life. *Eye*, *24*(1), 29–35. https://doi.org/10.1038/eye.2009.56

Gall, C., Brösel, D., & Sabel, B. A. (2013). Remaining visual field and preserved subjective visual functioning prevent mental distress in patients with visual field defects. *Frontiers in Human Neuroscience*, *7*(9), 1–8. https://doi.org/10.3389/fnhum.2013.00584

Garweg, J. G., Stefanickova, J., Hoyng, C., Schmelter, T., Niesen, T., Sowade, O., Sivaprasad, S., Adan, A., Alexik, M., Ali, F., Amaro, M., Balciuniene, V. J., Bandello, F. M., Arias Barquet, L., Beck, A., Bell, K., Boscia, F., Bures, A., Carneiro, Â., … Zarnowski, T. (2019). Vision-Related Quality of Life in Patients with Diabetic Macular Edema Treated with Intravitreal Aflibercept: The AQUA Study. *Ophthalmology Retina*, *3*(7), 567–575. https://doi.org/10.1016/j.oret.2019.03.012

Gazzard, G., Kolko, M., Iester, M., Crabb, D. P., Emesz, M., Hirn, C., Hommer, A., Kaya, S., Kellner, L., Lenzhofer, M., Vass, C., Collignon, N., de Groot, V., Duchesne, B., Kestelyn, P., Koppen, C., Stalmans, I., Stevens, A. M., Samsonova, B., … Théa, L. (2021). A scoping review of quality of life questionnaires in glaucoma patients. In *Journal of Glaucoma*30, (8), 732–743. https://doi.org/10.1097/IJG.0000000000001889

Girdler, S. J., Boldy, D. P., Dhaliwal, S. S., Crowley, M., & Packer, T. L. (2010). Vision self-management for older adults: A randomised controlled trial. *British Journal of Ophthalmology*, *94*(2), 223–228. https://doi.org/10.1136/bjo.2008.147538

Glick, P., Luoto, J., Orrs, M. S., Oliva, M. S., Tabin, G. C., Sanders, D. S., Thomas, B. J., Ruit, S., Belachew, T., & Tasfaw, A. K. (2019). The individual and household impacts of cataract surgery on older blind adults in ethiopia. *Ophthalmic Epidemiology*, *26*(1), 7–18. https://doi.org/10.1080/09286586.2018.1504310

Globe, D. R., Levin, S., Chang, T. S., & Mackenzie, P. J. (2002). Validity of the SF-12 Quality of Life. *October*, 1793–1798.

Goertz, Y. H. H., Houkes, I., & Bosma, H. (2017). Factors associated with participation on the competitive labour market of people with visual impairments in the Netherlands. *Work*, *58*(3), 251–261. https://doi.org/10.3233/WOR-172629

Goldberg, I., Clement, C. I., Chiang, T. H., Walt, J. G., Lee, L. J., Graham, S., & Healey, P. R. (2009). Assessing quality of life in patients with glaucoma using the glaucoma quality of life-15 (GQL-15) questionnaire. *Journal of Glaucoma*, *18*(1), 6–12. https://doi.org/10.1097/IJG.0b013e3181752c83

Goldstein, J. E., Chun, M. W., Fletcher, D. C., Deremeik, J. T., & Massof, R. W. (2014). Visual ability of patients seeking outpatient lowvision services in the united states. *JAMA Ophthalmology*, *132*(10), 1169–1177. https://doi.org/10.1001/jamaophthalmol.2014.1747

Gothwal, V. K., & Bagga, D. K. (2013). Vision and quality of life index: Validation of the Indian version using rasch analysis. *Investigative Ophthalmology and Visual Science*, *54*(7), 4871–4881. https://doi.org/10.1167/iovs.13-11892

Graham-Rowe, E., Lorencatto, F., Lawrenson, J. G., Burr, J. M., Grimshaw, J. M., Ivers, N. M., Presseau, J., Vale, L., Peto, T., Bunce, C., & J Francis, J. (2018). Barriers to and enablers of diabetic retinopathy screening attendance: a systematic review of published and grey literature. In *Diabetic Medicine,* 35, (10), 1308–1319). https://doi.org/10.1111/dme.13686

Grundler, W., & Strasburger, H. (2020). Visual attention outperforms visual-perceptual parameters required by law as an indicator of on-road driving performance. *PLoS ONE*, *15*(8). https://doi.org/10.1371/journal.pone.0236147

Guerette, A. R., & Smedema, S. M. (2011). The relationship of perceived social support with well-being in adults with visual impairments. *Journal of Visual Impairment and Blindness*, *105*(7), 425–439. https://doi.org/10.1177/0145482x1110500705

Habtamu, E., Wondie, T., Aweke, S., Tadesse, Z., Zerihun, M., Zewdie, Z., Callahan, K., Emerson, P. M., Kuper, H., Bailey, R. L., Mabey, D. C. W., Rajak, S. N., Polack, S., Weiss, H. A., & Burton, M. J. (2015). Trachoma and Relative Poverty: A Case-Control Study. *PLoS Neglected Tropical Diseases*, *9*(11). https://doi.org/10.1371/journal.pntd.0004228

Haegele, J. A., Famelia, R., & Lee, J. (2017). Health-related quality of life, physical activity, and sedentary behavior of adults with visual impairments. *Disability and Rehabilitation*, *39*(22), 2269–2276. https://doi.org/10.1080/09638288.2016.1225825

Harold E. Bedell, Jianliang Tong, Stanley Y. Woo, Jon R.House, & Tammy Nguyen. (2010). Orientation Discrimination with Macular Changes Associated with Early AMD. *Optom Vis Sci*, *86*(5), 485–491. https://doi.org/10.1097/OPX.0b013e31819fa6e2.Orientation

Hartong, D. T., & Kooijman, A. C. (2006). Night-vision goggles for night-blind subjects: Subjective evaluation after 2 years of use. *Ophthalmic and Physiological Optics*, *26*(5), 490–496. https://doi.org/10.1111/j.1475-1313.2006.00392.x

Hart, P. M., Stevenson, M. R., Montgomery, A. M., Muldrew, K. A., & Chakravarthy, U. (2005). Further validation of the Daily Living Tasks Dependent on Vision: Identification of domains. *British Journal of Ophthalmology*, *89*(9), 1127–1130. https://doi.org/10.1136/bjo.2004.059683

Havermaet, J. van, de Schauwer, E., & van Hove, G. (n.d.). *Unseen? A qualitative study on how mothers and fathers living with a visual impairment experience parenthood*.

Haymes, S. A., Johnston, A. W., & Heyes, A. D. (2002). Relationship between vision impairment and ability to perform activities of daily living. *Ophthalmic and Physiological Optics*, *22*(2), 79–91. https://doi.org/10.1046/j.1475-1313.2002.00016.x

He, D., Chen, X., Zhao, D., & Zhou, H. (2011). Cognitive function, depression, fatigue, and activities of daily living in patients with neuromyelitis optica after acute relapse. *International Journal of Neuroscience*, *121*(12), 677–683. https://doi.org/10.3109/00207454.2011.608456

Heyl, V., & Wahl, H. W. (2001). Psychosocial adaptation to age-related vision loss: A six-year perspective. *Journal of Visual Impairment and Blindness*, *95*(12), 739–748. https://doi.org/10.1177/0145482x0109501204

Hirneiß, C., Reznicek, L., Vogel, M., & Pesudovs, K. (2013). The impact of structural and functional parameters in glaucoma patients on patient-reported visual functioning. *PLoS ONE*, *8*(12), 1–7. https://doi.org/10.1371/journal.pone.0080757

Hirneiß, C., Vogel, M., Kampik, A., Neubauer, A. S., & Kernt, M. (2011). Messung der glaukomspezifischen Funktionsfähigkeit mit dem GQL-15 und Korrelation mit Funktionsparametern des Sehens. *Ophthalmologe*, *108*(10), 939–946. https://doi.org/10.1007/s00347-011-2402-1

Hirooka, K., Sato, S., Nitta, E., & Tsujikawa, A. (2016). The relationship between vision-related quality of life and visual function in glaucoma patients. *Journal of Glaucoma*, *25*(6), 505–509. https://doi.org/10.1097/IJG.0000000000000372

Hochberg, C., Maul, E., Chan, E. S., van Landingham, S., Ferrucci, L., Friedman, D. S., & Ramulu, P. Y. (2012). Association of vision loss in glaucoma and age-related macular degeneration with IADL disability. *Investigative Ophthalmology & Visual Science*, *53*(6), 3201–3206. https://doi.org/10.1167/iovs.12-9469

Hollands, H., Brox, A. C., Chang, A., Adilman, S., Chakraborti, B., Kliever, G., & Maberley, D. A. L. (2009). Correctable visual impairment and its impact on quality of life in a marginalized Canadian neighbourhood. *Canadian Journal of Ophthalmology*, *44*(1), 42–48. https://doi.org/10.3129/I08-167

Honaker, J. A., Tomasek, R., Bean, K., & Logan, B. (2012). Impact of visual disorders on vestibular and balance rehabilitation therapy outcomes in soldiers with blast injury. *International Tinnitus Journal*, *17*(2), 124–133. https://doi.org/10.5935/0946-5448.20120023

Hong, T., Mitchell, P., Burlutsky, G., Fong, C. S. U., Rochtchina, E., & Wang, J. J. (2013). Visual Impairment and subsequent use of support services among older people: Longitudinal findings from the Blue Mountains Eye Study. *American Journal of Ophthalmology*, *156*(2), 393-399.e1. https://doi.org/10.1016/j.ajo.2013.04.002

Horowitz, A., Brennan, M., Reinhardt, J. P., & MacMillan, T. (2006). The impact of assistive device use on disability and depression among older adults with age-related vision impairments. *Journals of Gerontology: Series B, Psychological Sciences and Social Sciences*, *61*(5), 274–280. https://doi.org/10.1093/geronb/61.5.S274

Horowitz, A., Reinhardt, J. P., & Boerner, K. (2005). The effect of rehabilitation on depression among visually disabled older adults. *Aging and Mental Health*, *9*(6), 563–570. https://doi.org/10.1080/13607860500193500

Houde, S. C., & Martha, A. (2003). Age-related vision loss in older adults: A challenge for gerontological nurses. *Journal of Gerontological Nursing*, *29*(4), 25–33.

Huang, W., Gao, K., Liu, Y., Liang, M., & Zhang, X. (2020). The Adverse Impact of Glaucoma on Psychological Function and Daily Physical Activity. *Journal of Ophthalmology*, *2020*. https://doi.org/10.1155/2020/9606420

Hu, C. X., Zangalli, C., Hsieh, M., Gupta, L., Williams, A. L., Richman, J., & Spaeth, G. L. (2014). What do patients with glaucoma see? Visual symptoms reported by patients with glaucoma. *American Journal of the Medical Sciences*, *348*(5), 403–409. https://doi.org/10.1097/MAJ.0000000000000319

Husin, M. H., & Lim, Y. K. (2020). InWalker: smart white cane for the blind. *Disability and Rehabilitation: Assistive Technology*, *15*(6), 701–707. https://doi.org/10.1080/17483107.2019.1615999

Ikeda, M. C., Bando, A. H., Hamada, K. U., Nakamura, V. P. L., Prata, T. S., Paranhos, A., Tatham, A. J., & Gracitelli, C. P. B. (2021). Is reading performance impaired in glaucoma patients with preserved central vision? *Journal of Glaucoma*, *30*(4), 153–158. https://doi.org/10.1097/IJG.0000000000001806

Iliffe, S., Kharicha, K., Harari, D., Swift, C., Gillmann, G., & Stuck, A. E. (2013). Health risk appraisal in older people 6: Factors associated with self-reported poor vision and uptake of eye tests in older people. *BMC Family Practice*, *14*, 2–7. https://doi.org/10.1186/1471-2296-14-130

Inoue, M., Arakawa, A., Yamane, S., & Kadonosono, K. (2014). Intravitreal injection of ranibizumab using a pro re nata regimen for age-related macular degeneration and vision-related quality of life. *Clinical Ophthalmology*, *8*, 1711–1716. https://doi.org/10.2147/OPTH.S68293

Islam, F. M. A., Kawasaki, R., & Finger, R. P. (2018). Factors associated with participation in a diabetic retinopathy screening program in a rural district in Bangladesh. *Diabetes Research and Clinical Practice*, *144*, 111–117. https://doi.org/10.1016/j.diabres.2018.08.012

Jacobs, J. M., Hammerman-Rozenberg, R., Maaravi, Y., Cohen, A., & Stessman, J. (2005). The impact of visual impairment on health, function and mortality. *Aging Clinical and Experimental Research*, *17*(4), 281–286. https://doi.org/10.1007/BF03324611

Jampel, H. D., Frick, K. D., Janz, N. K., Wren, P. A., Musch, D. C., Rimal, R., & Lichter, P. R. (2007). Depression and mood indicators in newly diagnosed glaucoma patients. *American Journal of Ophthalmology*, *144*(2). https://doi.org/10.1016/j.ajo.2007.04.048

Jason C.S. Leung, Timothy C.Y. Kwok, Dicken C.C. Chan, Kay W.K. Yuen, Anthony W.L. Kwok, Dicky T.K. Choy, Edith M.C. Lau, & P.C. Leung. (2012). Visual functioning and quality of life among the elderly in Hong Kong. *Int J Geriatr Psychiatry*, *27*(8), 807–815. https://doi.org/10.1002/gps.2789.Visual

Jayawant, S. S., Bhosle, M. J., Anderson, R. T., & Balkrishnan, R. (2007). Depressive symptomatology, medication persistence, and associated healthcare costs in older adults with glaucoma. *Journal of Glaucoma*, *16*(6), 513–520. https://doi.org/10.1097/IJG.0b013e31804a5ec6

Jeon, B. J., & Cha, T. H. (2013). The effects of balance of low vision patients on activities of daily living. *Journal of Physical Therapy Science*, *25*(6), 693–696. https://doi.org/10.1589/jpts.25.693

Jin, S., Tam, A. L. C., Chen, L., Trope, G. E., Buys, Y. M., & Jin, Y. P. (2019). Canadians with visual impairment utilize home care services more frequently. *Canadian Journal of Ophthalmology*, *54*(2), 196–202. https://doi.org/10.1016/j.jcjo.2018.03.007

Jin, Y. P., Buys, Y. M., Xiong, J., & Trope, G. E. (2013). Government-insured routine eye examinations and prevalence of nonrefractive vision problems among elderly. *Canadian Journal of Ophthalmology*, *48*(3), 167–172. https://doi.org/10.1016/j.jcjo.2013.01.002

Jin, Y. P., & Wong, D. T. (2008). Self-reported visual impairment in elderly Canadians and its impact on healthy living. *Canadian Journal of Ophthalmology*, *43*(4), 407–413. https://doi.org/10.3129/I08-077

Jones, G. C., Crews, J. E., & Danielson, M. L. (2010). Health risk profile for older adults with blindness: An application of the international classification of functioning, disability, and health framework. *Ophthalmic Epidemiology*, *17*(6), 400–410. https://doi.org/10.3109/09286586.2010.528137

Jones, P. R., Somoskeöy, T., Chow-Wing-Bom, H., & Crabb, D. P. (2020). Seeing other perspectives: evaluating the use of virtual and augmented reality to simulate visual impairments (OpenVisSim). *Npj Digital Medicine*, *3*(1). https://doi.org/10.1038/s41746-020-0242-6

Kabedi, N. N., Kayembe, D. L., & Mwanza, J. C. (2020). Vision-related quality of life, anxiety and depression in Congolese patients with polypoidal choroidal vasculopathy. *Seminars in Ophthalmology*, *35*(3), 156–163. https://doi.org/10.1080/08820538.2020.1774623

Kamelska, A. M., & Mazurek, K. (2015). The assessment of the quality of life in visually impaired people with different level of physical activity. *Physical Culture and Sport. Studies and Research*, *67*(1), 31–41. https://doi.org/10.1515/pcssr-2015-0001

Kandel, H., Nguyen, V., Piermarocchi, S., Ceklic, L., Teo, K., Arnalich‐Montiel, F., Miotto, S., Daien, V., Gillies, M. C., & Watson, S. L. (2022). Quality of life impact of eye diseases: a Save Sight Registries study. *Clinical & Experimental Ophthalmology*, *50*(4), 386–397. https://doi.org/10.1111/ceo.14050

Karadeniz Ugurlu, S., Kocakaya Altundal, A. E., & Altin Ekin, M. (2017). Comparison of visionrelated quality of life in primary open-angle glaucoma and dry-type age-related macular degeneration. *Eye (Basingstoke)*, *31*(3), 395–405. https://doi.org/10.1038/eye.2016.219

Karakus, S., Mathews, P. M., Agrawal, D., Henrich, C., Ramulu, P. Y., & Akpek, E. K. (2018). Impact of Dry Eye on prolonged reading. *Optometry and Vision Science*, *95*(12), 1105–1113. https://doi.org/10.1097/OPX.0000000000001303

Karlsson, E., Mäki-Torkko, E., Widén, S., Gustafsson, J., Manchaiah, V., Mahomed-Asmail, F., Swanepoel, D. W., Yerraguntla, K., & Granberg, S. (2021). Validation of the Brief International Classification of Functioning, Disability and Health (ICF) core set for hearing loss: an international multicentre study. *International Journal of Audiology*, *60*(6), 412–420. https://doi.org/10.1080/14992027.2020.1846088

Kawahara, Y., Deguchi, K., Hishikawa, N., Kurata, T., Sato, K., Kono, S., Omote, Y., Ohta, Y., Yamashita, T., & Abe, K. (2015). Cognitive and affective functions of aged subacute myelo-optico neuropathy patients in Japan. *Neurology and Clinical Neuroscience*, *3*(5), 173–178. https://doi.org/10.1111/ncn3.12000

Kay, S., & Ferreira, A. (2014). Mapping the 25-item national eye institute visual functioning questionnaire (NEI VFQ-25) to EQ-5D utility scores. *Ophthalmic Epidemiology*, *21*(2), 66–78. https://doi.org/10.3109/09286586.2014.888456

Kells, K. (2001). Ability of blind people to detect obstacles in unfamiliar environments. *Journal of Nursing Scholarship*, *33*(2), 153–157. https://doi.org/10.1111/j.1547-5069.2001.00153.x

Kempen, G. I. J. M., Ballemans, J., Ranchor, A. v., van Rens, G. H. M. B., & Zijlstra, G. A. R. (2012). The impact of low vision on activities of daily living, symptoms of depression, feelings of anxiety and social support in community-living older adults seeking vision rehabilitation services. *Quality of Life Research*, *21*(8), 1405–1411. https://doi.org/10.1007/s11136-011-0061-y

Kerr, N. M., Patel, H. Y., Chew, S. S., Ali, N. Q., Eady, E. K., & Danesh-Meyer, H. v. (2013). Patient satisfaction with topical ocular hypotensives. *Clinical and Experimental Ophthalmology*, *41*(1), 27–35. https://doi.org/10.1111/j.1442-9071.2012.02823.x

Khare, S., Rohatgi, J., Bhatia, M. S., & Dhaliwal, U. (2016). Burden and depression in primary caregivers of persons with visual impairment. *Indian Journal of Ophthalmology*, *64*(8), 572–577. https://doi.org/10.4103/0301-4738.191493

Khoo, K., Man, R. E. K., Rees, G., Gupta, P., Lamoureux, E. L., & Fenwick, E. K. (2019). The relationship between diabetic retinopathy and psychosocial functioning: a systematic review. In *Quality of Life Research,* 28(8), 2017–2039. https://doi.org/10.1007/s11136-019-02165-1

Khorrami-nejad, M., Sarabandi, A., Akbari, M.-R., & Askarizadeh, F. (2016). The impact of visual impairment on quality of life. *Journal Ophthalmology*, *5*(3), 96–103.

Kim, Y. S., Yi, M. Y., Hong, Y. J., & Park, K. H. (2018). The impact of visual symptoms on the quality of life of patients with early to moderate glaucoma. *International Ophthalmology*, *38*(4), 1531–1539. https://doi.org/10.1007/s10792-017-0616-1

Kirkman, M. A., Korsten, A., Leonhardt, M., Dimitriadis, K., de Coo, I. F., Klopstock, T., Griffiths, P. G., Hudson, G., Chinnery, P. F., & Yu-Wai-Man, P. (2009). Quality of life in patients with leber hereditary optic neuropathy. *Investigative Ophthalmology and Visual Science*, *50*(7), 3112–3115. https://doi.org/10.1167/iovs.08-3166

Kisanga, S. E., & Kisanga, D. H. (2020). The role of assistive technology devices in fostering the participation and learning of students with visual impairment in higher education institutions in Tanzania. *Disability and Rehabilitation: Assistive Technology*, *17*(7), 791–800. https://doi.org/10.1080/17483107.2020.1817989

Kowalski, J. W., Rentz, A. M., Walt, J. G., Lloyd, A., Lee, J., Young, T. A., Chen, W. H., Bressler, N. M., Lee, P., Brazier, J. E., Hays, R. D., & Revicki, D. A. (2012). Rasch analysis in the development of a simplified version of the national eye institute visual-function questionnaire-25 for utility estimation. *Quality of Life Research*, *21*(2), 323–334. https://doi.org/10.1007/s11136-011-9938-z

Kutzbach, B. R., Merrill, K. S., Hogue, K. M., Downes, S. J., Holleschau, A. M., MacDonald, J. T., & Summers, C. G. (2009). Evaluation of vision-specific quality-of-life in albinism. *Journal of AAPOS*, *13*(2), 191–195. https://doi.org/10.1016/j.jaapos.2008.10.008

Kuyk, T., Elliott, J. L., Wesley, J., Scilley, K., McIntosh, E., Mitchell, S., & Owsley, C. (2004). Mobility function in older veterans improves after blind rehabilitation. *Journal of Rehabilitation Research and Development*, *41*(3), 337–345. https://doi.org/10.1682/JRRD.2003.03.0038

Kuyk, T., Liu, L., Elliott, J. L., Grubbs, H. E., Owsley, C., McGwin, G., Griffin, R. L., & Fuhr, P. S. (2008). Health-related quality of life following blind rehabilitation. *Quality of Life Research*, *17*(4), 497–507. https://doi.org/10.1007/s11136-008-9336-3

Kymes, S. M. (2014). Is it time to move beyond the QALY in vision research? *Ophthalmic Epidemiology*, *21*(2), 63–65. https://doi.org/10.3109/09286586.2014.895843

Kymes, S. M., & Lee, B. S. (2007). Preference-Based Quality of Life Measures in people with visual impairment. *Optometry and Vision Science*, *84*(8), 809–816. https://doi.org/https://doi.org/10.1097/OPX.0b013e3181337638

Kymes, S. M., Walline, J. J., Zadnik, K., & Gordon, M. O. (2004). Quality of life in keratoconus. *American Journal of Ophthalmology*, *138*(4), 527–535. https://doi.org/10.1016/j.ajo.2004.04.031

la Grow, S. J. (2007). Predicting perceived quality of life scores from the National Eye Institute 25-item visual function questionnaire. *Optometry and Vision Science*, *84*(8), 785–788. https://doi.org/10.1097/OPX.0b013e31812f5f24

Laitinen, A., Sainio, P., Koskinen, S., Rudanko, S. L., Laatikainen, L., & Aromaa, A. (2007). The association between visual acuity and functional limitations: Findings from a nationally representative population survey. *Ophthalmic Epidemiology*, *14*(6), 333–342. https://doi.org/10.1080/01658100701473713

Lam, J., Robertson, K., Robertson, W., & Bernstein, R. (2015). Improving access to vision care for people who are homeless through eyeglasses recycling. *Journal of Health Care for the Poor and Underserved*, *26*(4), 1359–1367. https://doi.org/10.1353/hpu.2015.0123

Lamoureux, E., Gadgil, S., Pesudovs, K., Keeffe, J., Fenwick, E., Dirani, M., Salonen, S., & Rees, G. (2010). The relationship between visual function, duration and main causes of vision loss and falls in older people with low vision. *Graefe’s Archive for Clinical and Experimental Ophthalmology*, *248*(4), 527–533. https://doi.org/10.1007/s00417-009-1260-x

Latham, K., Baranian, M., Timmis, M., & Pardhan, S. (2015). Emotional health of people with visual impairment caused by retinitis pigmentosa. *PLoS ONE*, *10*(12). https://doi.org/10.1371/journal.pone.0145866

Lau, J. T. F., Lee, V., Fan, D., Lau, M., & Michon, J. (2004). Attitudes towards and perceptions of visual loss and its causes among Hong Kong Chinese adults. *Clinical and Experimental Ophthalmology*, *32*(3), 243–250. https://doi.org/10.1111/j.1442-9071.2004.00811.x

Lepl, A., Fran, J., Diakit, B., Tour, O., & Ecosse, E. (2020). A new condition specific quality of life measure for the blind and the partially sighted in Sub-Saharan Africa, the IOTAQOL: methodological aspects of the development procedure. *Quality of Life Research : An International Journal of Quality of Life Aspects of Treatment, Care and Rehabilitation*, *15*(8), 1373–1382. https://doi.org/10.1007/slll36-006-0023-y

Le, Q., Chen, Y., Wang, X., Li, Y., Hong, J., & Xu, J. (2011). Vision-related quality of life in patients with ocular chemical burns. *Investigative Ophthalmology and Visual Science*, *52*(12), 8951–8956. https://doi.org/10.1167/iovs.11-8355

Lethbridge, E. M., & Muldoon, C. (2018). Development of a Mobility-Related Quality-of-Life Measure for Individuals with Vision Impairments. *Journal of Visual Impairment & Blindness*, *112*(2), 169–181. https://doi.org/https://doi.org/10.1177/0145482X1811200205

Levinson, R. D., Monnet, D., Yu, F., Holland, G. N., Gutierrez, P., & Brezin, A. P. (2009). Longitudinal cohort study of patients with Birdshot Chorioretinopathy. V. quality of life at baseline. *American Journal of Ophthalmology*, *147*(2), 346–350. https://doi.org/10.1016/j.ajo.2008.08.011

Lewis, S. (2006). A closer look at the population of individuals with visual impairment in Florida. *Journal of Rehabilitation*, *72*(3), 26–32.

Leys, A., Zlateva, G., Shah, S. N., & Patel, M. (2008). Quality of life in patients with age-related macular degeneration: Results from the VISION study. *Eye*, *22*(6), 792–798. https://doi.org/10.1038/sj.eye.6702900

Li, K., Kou, J., Lam, Y., Lyons, P., & Nguyen, S. (2019). First-time experience in owning a dog guide by older adults with vision loss. *Journal of Visual Impairment and Blindness*, *113*(5), 452–463. https://doi.org/10.1177/0145482X19868351

Lim, N. C. S., Fan, C. H. J., Yong, M. K. H., Wong, E. P. Y., & Yip, L. W. Y. (2016). Assessment of Depression, Anxiety, and Quality of Life in Singaporean Patients with Glaucoma. *Journal of Glaucoma*, *25*(7), 605–612. https://doi.org/10.1097/IJG.0000000000000393

Lin, I. C., Lee, C. C., & Liao, S. L. (2015). Assessing quality of life in Taiwanese patients with Graves’ ophthalmopathy. *Journal of the Formosan Medical Association*, *114*(11), 1047–1054. https://doi.org/10.1016/j.jfma.2013.12.002

Lin, J. C., & Chie, W. C. (2010). Psychometric validation of the Taiwan Chinese version of the 25-Item National Eye Institute Visual Functioning Questionnaire. *Journal of Evaluation in Clinical Practice*, *16*(3), 619–626. https://doi.org/10.1111/j.1365-2753.2009.01253.x

Liu, C. J., & Chang, M. C. (2020). Interventions within the scope of occupational therapy practice to improve performance of daily activities for older adults with low vision: A systematic review. *American Journal of Occupational Therapy*, 74(1), 7401185010p1-7401185010p18. https://doi.org/10.5014/ajot.2020.038372

Li, Z., Cui, H., Zhang, L., Liu, P., & Yang, H. (2009). Cataract blindness and surgery among the elderly in rural southern harbin, China. *Ophthalmic Epidemiology*, *16*(2), 78–83. https://doi.org/10.1080/09286580802573193

Lord, S. R., & Dayhew, J. (2001). Visual risk factors for falls in older people. *Journal of the American Geriatrics Society*, *49*(5), 508–515. https://doi.org/https://doi.org/10.1046/j.1532-5415.2001.49107.x

Lynch, K. A. (2013). Survey reveals myths and misconceptions abundant among hiring managers about the capabilities of people who are visually impaired. *Journal of Visual Impairment and Blindness*, *107*(6), 408–410. https://doi.org/10.1177/0145482x1310700603

Mabuchi, F., Yoshimura, K., Kashiwagi, K., Yamagata, Z., Kanba, S., Iijima, H., & Tsukahara, S. (2012). Risk factors for anxiety and depression in patients with glaucoma. *British Journal of Ophthalmology*, *96*(6), 821–825. https://doi.org/10.1136/bjophthalmol-2011-300910

Machado, L. F., Kawamuro, M., Portela, R. C., Fares, N. T., Bergamo, V., de Souza, L. M., Paranhos, A., Prata, T. S., & Gracitelli, C. P. B. (2019). Factors associated with vision-related quality of life in Brazilian patients with glaucoma. *Arquivos Brasileiros de Oftalmologia*, *82*(6), 463–470. https://doi.org/10.5935/0004-2749.20190089

Maclennan, P. A., McGwin, G., Searcey, K., & Owsley, C. (2014). A survey of Alabama eye care providers in 2010-2011. *BMC Ophthalmology*, *14*(1), 1–10. https://doi.org/10.1186/1471-2415-14-44

Macnaughton, J., Latham, K., & Vianya-Estopa, M. (2019). Rehabilitation needs and activity limitations of adults with a visual impairment entering a low vision rehabilitation service in England. *Ophthalmic and Physiological Optics*, *39*(2), 113–126. https://doi.org/10.1111/opo.12606

Mahdaviazad, H., Bamdad, S., Roustaei, N., & Mohaghegh, S. (2018). Vision-Related Quality of Life in Iranian Patients With Keratoconus: National Eye Institute Vision Function Questionnaire-25. *Eye & Contact Lens*, *44*(6), S350–S354. https://doi.org/10.1097/ICL.0000000000000492

Majerníková, L., Hudáková, A., Obrocníková, A., Halász, B. G., & Kašcáková, M. (2021). Quality of life of patients with glaucoma in slovakia. *International Journal of Environmental Research and Public Health*, *18*(2), 1–13. https://doi.org/10.3390/ijerph18020485

Marahrens, L., Kern, R., Ziemssen, T., Fritsche, A., Martus, P., Ziemssen, F., & Roeck, D. (2017). Patients’ preferences for involvement in the decision-making process for treating diabetic retinopathy. *BMC Ophthalmology*, *17*(1). https://doi.org/10.1186/s12886-017-0526-z

Marella, M., Yu, M., Paudel, P., Michael, A., Ryan, K., Yasmin, S., & Minto, H. (2017). The situation of low vision services in Papua New Guinea: an exploratory study. *Clinical and Experimental Optometry*, *100*(1), 54–60. https://doi.org/10.1111/cxo.12446

Margolis, M. K., Coyne, K., Kennedy-Martin, T., Baker, T., Schein, O., & Revicki, D. A. (2002). Vision-specific instruments for the assessment of health-related quality of life and visual functioning: A literature review. *Pharmaco Economics*, *20*(12), 791–812. https://doi.org/10.2165/00019053-200220120-00001

Marques, A. P., Macedo, A. F., Lima Ramos, P., Moreno, L. H., Butt, T., Rubin, G., & Santana, R. (2019). Productivity Losses and Their Explanatory Factors Amongst People with Impaired Vision. *Ophthalmic Epidemiology*, *26*(6), 378–392. https://doi.org/10.1080/09286586.2019.1632904

Massof, R. W. (2014). A general theoretical framework for interpreting patient-reported outcomes estimated from ordinally scaled item responses. *Statistical Methods in Medical Research*, *23*(5), 409–429. https://doi.org/10.1177/0962280213476380

Massof, R. W., & Rubin, G. S. (2001). Visual function assessment questionnaires. *Survey of Ophthalmology*, *45*(6), 531–548. https://doi.org/10.1016/S0039-6257(01)00194-1

Mathew, R. S., Delbaere, K., Lord, S. R., Beaumont, P., Vaegan, & Madigan, M. C. (2011). Depressive symptoms and quality of life in people with age- related macular degeneration. *Ophthalmic and Physiological Optics*, *31*(4), 375–380. https://doi.org/10.1111/j.1475-1313.2011.00848.x

McKean-Cowdin R et al, 2008. (2008). Impact of Visual field loss on HRQOL in glaucoma. *Opthalmology*, *115*(6), 941–948. https://doi.org/10.1016/j.ophtha.2007.08.037.Impact

Mednick, Z., Jaidka, A., Nesdole, R., & Bona, M. (2017). Assessing the iPad as a tool for low-vision rehabilitation. *Canadian Journal of Ophthalmology*, *52*(1), 13–19. https://doi.org/10.1016/j.jcjo.2016.05.015

Miao, X., Shi, Z., Chen, H., Zhou, H., & Yang, R. (2017). The impact of pain, anxiety and depression on sleep quality in Chinese patients with neuromyelitis optica spectrum disorders. *Neurology Asia*, *22*(3), 235–241.

Mitchell, J., Wolffsohn, J., Woodcock, A., Anderson, S. J., Ffytche, T., Rubinstein, M., Amoaku, W., & Bradley, C. (2008). The MacDQoL Individualized Measure of the Impact of Macular Degeneration on Quality of Life: Reliability and Responsiveness. *American Journal of Ophthalmology*, *146*(3). https://doi.org/10.1016/j.ajo.2008.04.031

Moher, D., Liberati, A., Tetzlaff, J., Altman, D. G., Altman, D., Antes, G., Atkins, D., Barbour, V., Barrowman, N., Berlin, J. A., Clark, J., Clarke, M., Cook, D., D’Amico, R., Deeks, J. J., Devereaux, P. J., Dickersin, K., Egger, M., Ernst, E., … Tugwell, P. (2009). Preferred reporting items for systematic reviews and meta-analyses: The PRISMA statement. *PLoS Medicine*, *6*(7). https://doi.org/10.1371/journal.pmed.1000097

Mohler, A. J., Neufeld, P., & Perlmutter, M. S. (2015). Factors affecting readiness for low vision interventions in older adults. *American Journal of Occupational Therapy*, *69*(4). https://doi.org/10.5014/ajot.2015.014241

Morales, L., Varma, R., Paz, S., Lai, M., Mazhar, K., Andersen, R., Azen, S., & Los Angeles Latino Eye Study Group. (2010). Self-Reported Utilization of Eye Care among Latinos: The LosAngeles Latino Eye Study (LALES). *Ophthalmology*, *117*(2), 207–2015. https://doi.org/10.1016/j.ophtha.2009.07.015.Self-Reported

Morjaria, R., Alexander, I., Purbrick, R. M. J., Safa, R., Chong, N. V., Wulff, K., Foster, R. G., & Downes, S. M. (2019). Impact of diabetic retinopathy on sleep, mood, and quality of life. *Investigative Ophthalmology and Visual Science*, *60*(6), 2304–2310. https://doi.org/10.1167/iovs.18-26108

Muhammad, N., Alhassan, M., & Umar, M. (2015). Visual function and vision-related quality of life in presbyopic adult population of Northwestern Nigeria. *Nigerian Medical Journal*, *56*(5), 317. https://doi.org/10.4103/0300-1652.170379

Muir, K. W., Santiago-Turla, C., Stinnett, S. S., Herndon, L. W., Allingham, R. R., Challa, P., & Lee, P. P. (2008). Health literacy and vision-related quality of life. *British Journal of Ophthalmology*, *92*(6), 779–782. https://doi.org/10.1136/bjo.2007.134452

Murro, V., Sodi, A., Giacomelli, G., Mucciolo, D. P., Pennino, M., Virgili, G., & Rizzo, S. (2017). Reading ability and quality of life in stargardt disease. *European Journal of Ophthalmology*, *27*(6), 740–745. https://doi.org/10.5301/ejo.5000972

Murthy, G. V. S., Gupta, S. K., Dada, V. K., Pant, T. D., Savita, C., Sanga, L., & Neena, J. (2001). The use of a newspaper insertion to promote DIY testing of vision in India. *British Journal of Ophthalmology*, *85*(8), 952–955. https://doi.org/10.1136/bjo.85.8.952

Murthy, G. V. S., Schmidt, E., Gilbert, C., Edussuriya, K., & Pant, H. B. (2018). Impact of blindness, visual impairment and cataract surgery on quality of life and visual functioning among adults aged 40 years and above in Sri Lanka. *Ceylon Medical Journal*, *63*(5), 26. https://doi.org/10.4038/cmj.v63i5.8739

Nah, Y. S., Seong, G. J., & Kim, C. Y. (2002). Visual function and quality of life in Korean patients with glaucoma. In *Korean journal of ophthalmology*, 16 (2), 70–74. https://doi.org/10.3341/kjo.2002.16.2.70

Nastasi, J. A. (2020). Occupational therapy interventions supporting leisure and social participation for older adults with low vision: A systematic review. In *American Journal of Occupational Therapy*, 74(1). https://doi.org/10.5014/ajot.2020.038521

Nickels, S., Schuster, A. K., Singer, S., Wild, P. S., Laubert-Reh, D., Schulz, A., Finger, R. P., Michal, M., Beutel, M. E., Münzel, T., Lackner, K. J., & Pfeiffer, N. (2017). The National Eye Institute 25-Item Visual Function Questionnaire (NEI VFQ-25) - reference data from the German population-based Gutenberg Health Study (GHS). *Health and Quality of Life Outcomes*, *15*(1). https://doi.org/10.1186/s12955-017-0732-7

Nishijima, Daniel; K. Simel, David L; Wisner, David H; Holmes, J. F. (2016). Characterizing functional complaints in patients seeking outpatient Low vision services in the united states. *Physiology & Behavior*, *176*(1), 139–148. https://doi.org/10.1016/j.physbeh.2017.03.040

Noran N. Hairi, Awang Bulgiba, Robert G. Cumming, Vasi Naganathan, & Izzuna Mudla. (2011). Depressive symptoms, visual impairment and its influence on physical disability and functional limitation. *JAGS*, *59*(3), 557–559.

O’Connor, P. M., Scarr, B. C., Lamoureux, E. L., le Mesurier, R. T., & Keeffe, J. E. (2010). Validation of a quality of life questionnaire in the Pacific Island. *Ophthalmic Epidemiology*, *17*(6), 378–386. https://doi.org/10.3109/09286586.2010.528134

Odberg, T., Jakobsen, J. E., Hultgren, S. J., & Halseide, R. (2001). The impact of glaucoma on the quality of life of patients in Norway. *Acta Ophthalmologica Scandinavica*, *79*(2), 116–120. https://doi.org/10.1034/j.1600-0420.2001.079002116.x

Okamoto, M., Sugisaki, K., Murata, H., Hirasawa, H., Mayama, C., & Asaoka, R. (2014). Impact of better and worse eye damage on quality of life in advanced glaucoma. *Scientific Reports*, *4*, 1–6. https://doi.org/10.1038/srep04144

Omar, R., Rahman, M. H. A., Knight, V. F., Mustaphal, M., & Mohammed, Z. (2014). Mental health state and quality of life questionnaire in low vision assessment: A case report. *BMC Research Notes*, *7*(1), 5–8. https://doi.org/10.1186/1756-0500-7-667

Order, S., Phillips, G., O’Mahony, L., & Sturgess, K. (2021). The Vision Impaired as a Radio Audience: Meeting Their Audio Needs in the 21st Century. *Journal of Radio and Audio Media*, *28*(1), 107–124. https://doi.org/10.1080/19376529.2020.1854758

Orton, E., Forbes-Haley, A., Tunbridge, L., & Cohen, S. (2013). Equity of uptake of a diabetic retinopathy screening programme in a geographically and socio-economically diverse population. *Public Health*, *127*(9), 814–821. https://doi.org/10.1016/j.puhe.2013.04.015

Osaba, M., Doro, J., Liberal, M., Lagunas, J., Kuo, I. C., Víctor, ;, & Reviglio, E. (2019). Relationship Between Legal Blindness and Depression. *Discovery &Innovation Ophthalmology Journal Med Hypothesis Discov Innov Ophthalmol*, *8*(4), 306–311.

Owen, C. G., Rudnicka, A. R., Smeeth, L., Evans, J. R., Wormald, R. P. L., & Fletcher, A. E. (2006). Is the NEI-VFQ-25 a useful tool in identifying visual impairment in an elderly population? *BMC Ophthalmology*, *24*, 1–9. https://doi.org/10.1186/1471-2415-6-24

Owsley, C., & McGwin, G. (2004). Depression and the 25-item National Eye Institute Visual Function Questionnaire in older adults. *Ophthalmology*, *111*(12), 2259–2264. https://doi.org/10.1016/j.ophtha.2004.06.026

Owsley, C., McGwin, G., Scilley, K., Dreer, L. E., Bray, C. R., & Mason, J. O. (2006). Focus groups with persons who have age-related macular degeneration: Emotional issues. *Rehabilitation Psychology*, *51*(1), 23–29. https://doi.org/10.1037/0090-5550.51.1.23

Pache, M., & Flammer, J. (2006). A Sick Eye in a Sick Body? Systemic Findings in Patients with Primary Open-angle Glaucoma. *Survey of Ophthalmology*, *51*(3), 179–212. https://doi.org/10.1016/j.survophthal.2006.02.008

Palagyi, A., Ng, J. Q., Rogers, K., Meuleners, L., McCluskey, P., White, A., Morlet, N., & Keay, L. (2017). Fear of falling and physical function in older adults with cataract: Exploring the role of vision as a moderator. *Geriatrics and Gerontology International*, *17*(10), 1551–1558. https://doi.org/10.1111/ggi.12930

Paletta Guedes, R. A., Paletta Guedes, V. M., Freitas, S. M., & Chaoubah, A. (2014). Utility values for glaucoma in Brazil and their correlation with visual function. *Clinical Ophthalmology*, *8*, 529–535. https://doi.org/10.2147/OPTH.S60105

Palmer, S. L., Winskell, K., Patterson, A. E., Boubacar, K., Ibrahim, F., Namata, I., Oungoila, T., Kané, M. S., Hassan, A. S., Mosher, A. W., Hopkins, D. R., & Emerson, P. M. (2014). “A living death”: A qualitative assessment of quality of life among women with trichiasis in rural Niger. *International Health*, *6*(4), 291–297. https://doi.org/10.1093/inthealth/ihu054

Pancotto, H. P., Tome, C. A., & Esteves, A. M. (2021). Influence of swimming on sleep and quality of life of people with visual impairments. *Revista Brasileira de Medicina Do Esporte*, *27*(2), 179–183. https://doi.org/10.1590/1517-869220212702191748

Pan, C. W., Liu, H., Sun, H. P., & Xu, Y. (2015). Increased difficulties in managing stairs in visually impaired older adults: A community-based survey. In *PLoS ONE*, 10(11). https://doi.org/10.1371/journal.pone.0142516

Pandey, S., Lin, Y., Collier-Tenison, S., & Bodden, J. (2012). Social factors determining the experience of blindness among pregnant women in developing countries: The case of India. *Health and Social Work*, *37*(3), 157–169. https://doi.org/10.1093/hsw/hls025

Papageorgiou, E., Hardiess, G., Schaeffel, F., Wiethoelter, H., Karnath, H. O., Mallot, H., Schoenfisch, B., & Schiefer, U. (2007). Assessment of vision-related quality of life in patients with homonymous visual field defects. *Graefe’s Archive for Clinical and Experimental Ophthalmology*, *245*(12), 1749–1758. https://doi.org/10.1007/s00417-007-0644-z

Parameswarappa, D. C., Maltsev, D. S., Goud, A., Singh, S. R., & Chhablani, J. (2021). Characteristics of central serous chorioretinopathy without leakage. *Journal of Current Ophthalmology*, *33*(2), 152–157. https://doi.org/10.1016/j.joco.2019.09.006

Park, H. W., Lee, W., & Yoon, J. H. (2018). Gender-related effects of vision impairment characteristics on depression in Korea. *Ophthalmic Epidemiology*, *25*(2), 105–112. https://doi.org/10.1080/09286586.2017.1361453

Párraga, I., López-Torres, J., Navarro, B., Andrés, F., Escobar, F., & López, Á. (2012). The reliability and validity of the daily activities dependence on vision (DADV) scale to evaluate vision related functional limitations. *Archives of Gerontology and Geriatrics*, *55*(1), 120–125. https://doi.org/10.1016/j.archger.2011.06.001

Patnaik, J. L., Pecen, P. E., Hanson, K., Lynch, A. M., Cathcart, J. N., Siringo, F. S., Mathias, M. T., & Mandava, N. (2019). Driving and Visual Acuity in Patients with Age-Related Macular Degeneration. *Ophthalmology Retina*, *3*(4), 336–342. https://doi.org/10.1016/j.oret.2018.11.004

Patrick Yeatts, R. (2005). Quality of life in patients with graves ophthalmopathy. *American Ophthalmological Society*, *103*, 368–411. https://doi.org/10.1016/j.ajo.2006.04.012

Pattaramongkolrit, S., Sindhu, S., Thosigha, O., & Somboontanot, W. (2013). Fall-related Factors among Older, Visually-Impaired Thais. *Pacific Rim International Journal of Nursing Research*, *17*(2), 181–196.

Patty, N. J. S., Koopmanschap, M., & Holtzer-Goor, K. (2018). A cost-effectiveness study of ICT training among the visually impaired in the Netherlands. *BMC Ophthalmology*, *18*(1), 1–10. https://doi.org/10.1186/s12886-018-0761-y

Pearce, E., Crossland, M. D., & Rubin, G. S. (2011). The efficacy of low vision device training in a hospital-based low vision clinic. *British Journal of Ophthalmology*, *95*(1), 105–108. https://doi.org/10.1136/bjo.2009.175703

Peric, S., Stojanovic, V. R., Basta, I., Peric, M., Milicev, M., Pavlovic, S., & Lavrnic, D. (2013). Influence of multisystemic affection on health-related quality of life in patients with myotonic dystrophy type 1. *Clinical Neurology and Neurosurgery*, *115*(3), 270–275. https://doi.org/10.1016/j.clineuro.2012.05.015

Pesudovs, K., Garamendi, E., Keeves, J. P., & Elliott, D. B. (2003). The Activities of Daily Vision Scale for cataract surgery outcomes: Re-evaluating validity with Rasch analysis. *Investigative Ophthalmology and Visual Science*, *44*(7), 2892–2899. https://doi.org/10.1167/iovs.02-1075

Peterson, K. M., Huisingh, C. E., Girkin, C., Owsley, C., & Rhodes, L. A. (2018). Patient satisfaction with care in an urban tertiary referral academic glaucoma clinic in the us. *Patient Preference and Adherence*, *12*, 775–781. https://doi.org/10.2147/PPA.S162439

Peters, T., Klingberg, S., Zrenner, E., & Wilhelm, B. (2013). Emotional wellbeing of blind patients in a pilot trial with subretinal implants. *Graefe’s Archive for Clinical and Experimental Ophthalmology*, *251*(6), 1489–1493. https://doi.org/10.1007/s00417-012-2210-6

Petrillo, J., Cano, S. J., McLeod, L. D., & Coon, C. D. (2015). Using classical test theory, item response theory, and rasch measurement theory to evaluate patient-reported outcome measures: A comparison of worked examples. *Value in Health*, *18*(1), 25–34. https://doi.org/10.1016/j.jval.2014.10.005

Pilling, R. F., Thompson, J. R., & Gottlob, I. (2005). Social and visual function in nystagmus. *British Journal of Ophthalmology*, *89*(10), 1278–1281. https://doi.org/10.1136/bjo.2005.070045

Ponchillia, P. E., Strause, B., & Ponchillia, S. v. (2002). Athletes with visual impairments: Attributes and sports participation. *Journal of Visual Impairment and Blindness*, *96*(4), 267–272. https://doi.org/10.1177/0145482x0209600408

Pondorfer, S. G., Terheyden, J. H., Overhoff, H., Stasch-Bouws, J., Holz, F. G., & Finger, R. P. (2021). Development of the vision impairment in low luminance questionnaire. *Translational Vision Science and Technology*, *10*(1), 1–11. https://doi.org/10.1167/tvst.10.1.5

Popescu, M. L., Boisjoly, H., Schmaltz, H., Kergoat, M. J., Rousseau, J., Moghadaszadeh, S., Djafari, F., & Freeman, E. E. (2012). Explaining the relationship between three eye diseases and depressive symptoms in older adults. *Investigative Ophthalmology & Visual Science*, *53*(4), 2308–2313. https://doi.org/10.1167/iovs.11-9330

Prem Kumar, S. G., Mondal, A., Vishwakarma, P., Kundu, S., Lalrindiki, R., & Kurian, E. (2018). Factors limiting the Northeast Indian elderly population from seeking cataract surgical treatment: Evidence from Kolasib district, Mizoram, India. *Indian Journal of Ophthalmology*, *66*(7), 969–974. https://doi.org/10.4103/ijo.IJO_1184_17

Prior, M., Ramsay, C. R., Burr, J. M., Campbell, S. E., Jenkinson, D. J., Asoaka, R., & Francis, J. J. (2013). Theoretical and empirical dimensions of the Aberdeen Glaucoma Questionnaire: A cross sectional survey and principal component analysis. *BMC Ophthalmology*, *13*(1), 1–11. https://doi.org/10.1186/1471-2415-13-72

Puhan, M. A., Ahuja, A., Van Natta, M. L., Ackatz, L. E., & Meinert, C. (2011). Interviewer versus self-administered health-related quality of life questionnaires. Does it matter? *Health and Quality of Life Outcomes*, *9*, 1–11. https://doi.org/10.1186/1477-7525-9-30

Qiu, M., & Shields, C. L. (2015). Choroidal nevus in the United States adult population racial disparities and associated factors in the national health and nutrition examination survey. *Ophthalmology*, *122*(10), 2071–2083. https://doi.org/10.1016/j.ophtha.2015.06.008

Qiu, M., Wang, S. Y., Singh, K., & Lin, S. C. (2013). Association between myopia and glaucoma in the united states population. *Investigative Ophthalmology and Visual Science*, *54*(1), 830–835. https://doi.org/10.1167/iovs.12-11158

Quaranta, L., Riva, I., Gerardi, C., Oddone, F., Floriano, I., & Konstas, A. G. P. (2016). Quality of Life in Glaucoma: A Review of the Literature. *Advances in Therapy*, *33*(6), 959–981. https://doi.org/10.1007/s12325-016-0333-6

Rafaely, L., Carmel, S., & Bachner, Y. G. (2018). Subjective well-being of visually impaired older adults living in the community. *Aging and Mental Health*, *22*(9), 1223–1231. https://doi.org/10.1080/13607863.2017.1341469

Ramulu, P. Y., Mihailovic, A., West, S. K., Gitlin, L. N., & Friedman, D. S. (2019). Predictors of Falls per Step and Falls per Year At and Away From Home in Glaucoma. *American Journal of Ophthalmology*, *200*, 169–178. https://doi.org/10.1016/j.ajo.2018.12.021

Ramulu, P. Y., West, S. K., Munoz, B., Jampel, H. D., & Friedman, D. S. (2009). Driving cessation and driving limitation in glaucoma. The Salisbury Eye evaluation project. *Ophthalmology*, *116*(10), 1846–1853. https://doi.org/10.1016/j.ophtha.2009.03.033

Ratanasukon, M., Tongsomboon, J., Bhurayanontachai, P., & Jirarattanasopa, P. (2016). The impact of vision impairment (IVI) questionnaire; validation of the Thai-version and the implementation on vision-related quality of life in Thai rural community. *PLoS ONE*, *11*(5). https://doi.org/10.1371/journal.pone.0155509

Ratchford, R. S., & Krause, A. (2004). Visually Impaired Older Adults and Home-Based Leisure Activities: The Effects of Person-Environment Congruence. *Journal of Visual Impairment and Blindness*, *98*(1), 14–27. https://doi.org/https://doi.org/10.1177/0145482X0409800103

Razavi, H., Baglin, E., Sharangan, P., Caruso, E., Tindill, N., Griffin, S., & Guymer, R. (2018). Gaming to improve vision: 21st century self-monitoring for patients with age-related macular degeneration. *Clinical and Experimental Ophthalmology*, *46*(5), 480–484. https://doi.org/10.1111/ceo.13097

Rebouças, C. B. de A., de Araújo, M. M., Braga, F. C., Fernandes, G. T., & Costa, S. C. (2016). Evaluation of quality of life of visually impaired. *Revista Brasileira de Enfermagem*, *69*(1), 64–70. https://doi.org/10.1590/0034-7167.2016690110i

Rees, G., Fenwick, E., Keeffe, J. E., Mellor, D., & Lamoureux, E. L. (2011). Managing depression in patients with vision impairment: A descriptive study of practitioners’ beliefs and confidence. *Australasian Journal on Ageing*, *30*(3), 130–135. https://doi.org/10.1111/j.1741-6612.2010.00467.x

Rees, G., Saw, C. L., Lamoureux, E. L., & Keeffe, J. E. (2007). Self-management programs for adults with low vision: Needs and challenges. *Patient Education and Counseling*, *69*(1–3), 39–46. https://doi.org/10.1016/j.pec.2007.06.016

Rees, G., Tee, H. W., Marella, M., Fenwick, E., Dirani, M., & Lamoureux, E. L. (2010). Vision-specific distress and depressive symptoms in people with vision impairment. *Investigative Ophthalmology and Visual Science*, *51*(6), 2891–2896. https://doi.org/10.1167/iovs.09-5080

Reeves, B. C., Langham, J., Walker, J., Grieve, R., Chakravarthy, U., Tomlin, K., Carpenter, J., Guerriero, C., & Harding, S. P. (2009). Verteporfin Photodynamic Therapy Cohort Study. Report 2: Clinical Measures of Vision and Health-Related Quality of Life. *Ophthalmology*, *116*(12), 2463–2470. https://doi.org/10.1016/j.ophtha.2009.10.031

Reighard, C. L., Pillai, M. R., Shroff, S., Spaeth, G. L., Schilling, S. G., Wizov, S. S., Stein, J. D., Robin, A. L., Raja, V., & Ehrlich, J. R. (2019). Glaucoma-Associated Visual Task Performance and Vision-Related Quality of Life in South India. *Ophthalmology Glaucoma*, *2*(5), 357–363. https://doi.org/10.1016/j.ogla.2019.06.002

Renaud, J., & Bédard, E. (2013). Depression in the elderly with visual impairment and its association with quality of life. *Clinical Interventions in Aging*, *8*, 931–943. https://doi.org/10.2147/CIA.S27717

Rhodes, L. A., Huisingh, C. E., McGwin, G., Girkin, C. A., & Owsley, C. (2019). Glaucoma Patient Knowledge, Perceptions, and Predispositions for Telemedicine. *Journal of Glaucoma*, *28*(6), 481–486. https://doi.org/10.1097/IJG.0000000000001238

Richardson, J., Iezzi, A., Peacock, S., Sinha, K., Khan, M., Misajon, R., & Keeffe, J. (2012). Utility weights for the vision-related assessment of quality of life (AQoL)-7D instrument. *Ophthalmic Epidemiology*, *19*(3), 172–182. https://doi.org/10.3109/09286586.2012.674613

Riddering, A. T. (2018). Visual impairment and factors associated with difficulties with daily tasks. In *Dissertation Abstracts International: Section B: The Sciences and Engineering*, 78(8).

Robinson, J. L., Braimah Avery, V., Chun, R., Pusateri, G., & Jay, W. M. (2017). Usage of Accessibility Options for the iPhone and iPad in a Visually Impaired Population. *Seminars in Ophthalmology*, *32*(2), 163–171. https://doi.org/10.3109/08820538.2015.1045151

Robinson, J. L., Liu, Y., & Chen, Q. (2021). Licensure and Driving Status among Visually Impaired Persons. *Optometry and Vision Science*, *98*(12), 1334–1339. https://doi.org/10.1097/OPX.0000000000001812

Roque, A. B., da Silva Borges, G. F., Abe, R. Y., de Souza, O. F., Machado, M. C., Ferreira, T., José, N. K., & de Vasconcellos, J. P. C. (2021). The effects of age-related macular degeneration on quality of life in a Brazilian population. *International Journal of Retina and Vitreous*, *7*(1). https://doi.org/10.1186/s40942-021-00290-z

Rossi, G. C. M., Tinelli, C., Pasinetti, G. M., Milano, G., & Bianchi, P. E. (2009). Dry eye syndrome-related quality of life in glaucoma patients. *European Journal of Ophthalmology*, *19*(4), 572–579. https://doi.org/10.1177/112067210901900409

Rovner, B. W., Casten, R. J., Hegel, M. T., Massof, R. W., Leiby, B. E., Ho, A. C., & Tasman, W. S. (2013). Improving function in age-related macular degeneration: A randomized clinical trial. *Ophthalmology*, *120*(8), 1649–1655. https://doi.org/10.1016/j.ophtha.2013.01.022

Saaddine, J. B., Venkat Narayan, K. M., & Vinicor, F. (2003). Vision loss: A public health problem? *Ophthalmology*, *110*(2), 253–254. https://doi.org/10.1016/S0161-6420(02)01839-0

Saboo, U. S., Amparo, F., Abud, T. B., Schaumberg, D. A., & Dana, R. (2015). Vision-Related Quality of Life in Patients with Ocular Graft-versus-Host Disease. *Ophthalmology*, *122*(8), 1669–1674. https://doi.org/10.1016/j.ophtha.2015.04.011

Salomon, J. A., Vos, T., & Murra, C. J. L. (2013). Disability weights for vision disorders in Global Burden of Disease study. *The Lancet*, *381*(9860), 23–24. https://doi.org/10.1016/S0140-6736(12)62131-X

Samnieng, P. (2015). Relationship of Nutritional Status with Oral Health Status in Visual Impairment. *Makara Journal of Health Research*, *19*(1). https://doi.org/10.7454/msk.v19i1.4597

Saunier, V., Mercier, A. E., Gaboriau, T., Malet, F., Colin, J., Fournié, P., Malecaze, F., & Touboul, D. (2017). Vision-related quality of life and dependency in French keratoconus patients: Impact study. *Journal of Cataract and Refractive Surgery*, *43*(12), 1582–1590. https://doi.org/10.1016/j.jcrs.2017.08.024

Scanlan, J. M., & Cuddeford, J. E. (2004). Low vision rehabilitation: A comparison of traditional and extended teaching programs. *Journal of Visual Impairment and Blindness*, *98*(10), 601–611. https://doi.org/10.1177/0145482x0409801005

Schakel, W., Bode, C., Elsman, E. B. M., van der Aa, H. P. A., de Vries, R., van Rens, G. H. M. B., & van Nispen, R. M. A. (2019). The association between visual impairment and fatigue: a systematic review and meta-analysis of observational studies. In *Ophthalmic and Physiological Optics*, 39(6), 399–413. https://doi.org/10.1111/opo.12647

Schakel, W., Bode, C., van de Ven, P. M., van der Aa, H. P. A., Hulshof, C. T. J., van Rens, G. H. M. B., & van Nispen, R. M. A. (2019). Understanding fatigue in adults with visual impairment: A path analysis study of sociodemographic, psychological and healthrelated factors. *PLoS ONE*, *14*(10). https://doi.org/10.1371/journal.pone.0224340

Schémann, J. F., Leplège, A., Keita, T., & Resnikoff, S. (2002). From visual function deficiency to handicap: Measuring visual handicap in Mali. *Ophthalmic Epidemiology*, *9*(2), 133–148. https://doi.org/10.1076/opep.9.2.133.1519

Schiffman M. Rhett, Jacobsen Gordon, & Whitcup M. Scott. (2002). Visual functioning and general health status in patients with uveitis. *Evidence-Based Eye Care*, *3*(2), 92–93. https://doi.org/10.1097/00132578-200204000-00015

Scilley, K., DeCarlo, D. K., Wells, J., & Owsley, C. (2004). Vision-specific health-related quality of life in age-related maculopathy patients presenting for low vision services. *Ophthalmic Epidemiology*, *11*(2), 131–146. https://doi.org/10.1076/opep.11.2.131.28159

Scilley, K., & Owsley, C. (2002). Vision-specific health-related quality of life: Content areas for nursing home residents. *Quality of Life Research*, *11*(5), 449–462. https://doi.org/10.1023/A:1015677501386

Scott, A. W., Bressler, N. M., Ffolkes, S., Wittenborn, J. S., & Jorkasky, J. (2016). Public attitudes about eye and vision health. *JAMA Ophthalmology*, *134*(10), 1111–1118. https://doi.org/10.1001/jamaophthalmol.2016.2627

Scott, I. U., Schein, O. D., Feuer, W. J., Folstein, M. F., & Bandeen-Roche, K. (2001). Emotional distress in patients with retinal disease. *American Journal of Ophthalmology*, *131*(5), 584–589. https://doi.org/10.1016/S0002-9394(01)00832-7

Selb M., Escorpizo R., Kostanjsek N., Stucki G., Üstün B., & Cieza A. (2015). A guide on how to develop an International Classification of Functioning, Disability and Health Core Set. *Eur J Phys Rehabil Med*, *51*(3), 105–117.

Shah, S. U., Pilli, S., Telander, D. G., Morse, L. S., & Park, S. S. (2013). Survey of patients with age-related macular degeneration: Knowledge and adherence to recommendations. *Canadian Journal of Ophthalmology*, *48*(3), 204–209. https://doi.org/10.1016/j.jcjo.2013.01.013

Sharma, S., & Oliver-Fernandez, A. (2004). Age-related macular degeneration and quality of life: How to interpret a research paper in health-related quality of life. *Current Opinion in Ophthalmology*, *15*(3), 227–231. https://doi.org/10.1097/01.icu.0000124082.88389.cf

Sharon L. Christ, D. Diane Zheng, Bonnielin K. Swenor, Byron L. Lam, Sheila K. West, Stacey L. Tannenbaum, Beatriz E. Muñoz, & David J. Lee. (2014). Longitudinal Relationships Among Visual Acuity, Daily Functional Status, and Mortality. *JAMA Ophthalmol*, *132*(12), 1400–1406. https://doi.org/10.1001/jamaophthalmol.2014.2847.Longitudinal

Shrestha, M. K., Guo, C. W., Maharjan, N., Gurung, R., & Ruit, S. (2014). Health literacy of common ocular diseases in Nepal. *BMC Ophthalmology*, *14*(1). https://doi.org/10.1186/1471-2415-14-2

Silva, M. R. da, Nobre, M. I. R. de S., Carvalho, K. M. de, & Montilha, R. de C. I. (2014). Visual impairment, rehabilitation and International Classification of Functioning, Disability and Health. *Revista Brasileira de Oftalmologia*, *73*(5), 291–301. https://doi.org/10.5935/0034-7280.20140063

Simons, K. (2008). Old age and the functional consequences of amblyopia. In *Journal of AAPOS*,12(5), 429–430. https://doi.org/10.1016/j.jaapos.2008.05.013

Skalicky, S. E., Fenwick, E., Martin, K. R., Crowston, J., Goldberg, I., & McCluskey, P. (2016). Impact of age-related macular degeneration in patients with glaucoma: understanding the patients’ perspective. *Clinical and Experimental Ophthalmology*, *44*(5), 377–387. https://doi.org/10.1111/ceo.12672

Skalicky, S. E., Goldberg, I., & McCluskey, P. (2012). Ocular surface disease and quality of life in patients with glaucoma. *American Journal of Ophthalmology*, *153*(1), 1–9. https://doi.org/10.1016/j.ajo.2011.05.033

Skalicky, S. E., McAlinden, C., Khatib, T., Anthony, L. M., Sim, S. Y., Martin, K. R., Goldberg, I., & McCluskey, P. (2016). Activity limitation in glaucoma: Objective assessment by the cambridge glaucoma visual function test. *Investigative Ophthalmology and Visual Science*, *57*(14), 6158–6166. https://doi.org/10.1167/iovs.16-19458

Slimani, H., Danti, S., Ptito, M., & Kupers, R. (2014). Pain perception is increased in congenital but not late onset blindness. *PLoS ONE*, *9*(9). https://doi.org/10.1371/journal.pone.0107281

Smallfield, S., Berger, S., Hillman, B., Saltzgaber, P., Giger, J., & Kaldenberg, J. (2017). Living with low vision: Strategies supporting daily activity. *Occupational Therapy in Health Care*, *31*(4), 312–328. https://doi.org/10.1080/07380577.2017.1384969

Smallfield, S., & Kaldenberg, J. (2020). Occupational therapy interventions to improve reading performance of older adults with low vision: A systematic review. *American Journal of Occupational Therapy*, 74(1). https://doi.org/10.5014/ajot.2020.038380

Smith, T. M., Thomas, K., & Dow, K. (2009). The effect of an educational program for persons with macular degeneration: A pilot study. *Journal of Visual Impairment and Blindness*, *103*(4), 234–240. https://doi.org/10.1177/0145482x0910300407

Sotimehin, A. E., Yonge, A. v., Mihailovic, A., West, S. K., Friedman, D. S., Gitlin, L. N., & Ramulu, P. Y. (2018). Locations, Circumstances, and Outcomes of Falls in Patients With Glaucoma. *American Journal of Ophthalmology*, *192*, 131–141. https://doi.org/10.1016/j.ajo.2018.04.024

Spaeth, G., Walt, J., & Keener, J. (2006). Evaluation of quality of life for patients with glaucoma. *American Journal of Ophthalmology*, *141*(1), 3–14. https://doi.org/10.1016/j.ajo.2005.07.075

Spandau, U. H. M., Wechsler, S., & Blankenagel, A. (2002). Testing night vision goggles in a dark outside environment. *Optometry and Vision Science*, *79*(1), 39–45. https://doi.org/10.1097/00006324-200201000-00011

Steinman, B. A. (2016). Health Outcomes Associated with Self-Reported Vision Impairment in Older Adults. *Journal of Visual Impairment & Blindness*, *110*(6), 385–398. https://doi.org/https://doi.org/10.1177/0145482X1611000602

Stelmack, J. (2001). Quality of Life of Low-Vision Patients and Outcomes of Low-Vision rehabilitation. *Optometry and Vision Science*, *78*(5), 335–342. https://doi.org/https://doi.org/10.1097/00006324-200105000-00017

Stelmack, J. A., Rosenbloom, A. A., Brenneman, C. S., & Stelmack, T. R. (2003). Patients’ perceptions of the need for low vision devices. *Journal of Visual Impairment and Blindness*, *97*(9), 521–535. https://doi.org/10.1177/0145482X0309700904

Stelmack, J. A., Tang, X. C., Wei, Y., & Massof, R. W. (2012). The effectiveness of low-vision rehabilitation in 2 cohorts derived from the veterans affairs low-vision intervention trial. *Archives of Ophthalmology*, *130*(9), 1162–1168. https://doi.org/10.1001/archophthalmol.2012.1820

Stevenson, M. R., Hart, P. M., Montgomery, A. M., McCulloch, D. W., & Chakravarthy, U. (2004). Reduced vision in older adults with age related macular degeneration interferes with ability to care for self and impairs role as carer. *British Journal of Ophthalmology*, *88*(9), 1125–1130. https://doi.org/10.1136/bjo.2003.032383

Stoianov, M., de Oliveira, M. S., dos Santos R. S, Mariana C. L, Ferreira, M. H., de Oliveira M., & Gualtieri, M. (2019). The impacts of abnormal color vision on people’s life: an integrative review. *Quality of Life Research*, 28(4), 855–862. https://doi.org/10.1007/s11136-018-2030-1

Sundelin, K., & Lundström, M. (2012). Outcome of capsulotomy in patients with low vision and posterior capsule opacification. *Acta Ophthalmologica*, *90*(3), 221–225. https://doi.org/10.1111/j.1755-3768.2010.01909.x

Suneeta Dubey, Harleen Bedi, M. B., Preeti Matah, Jigyasa Sahu, Saptarshi Mukherjee, & Lokesh Chauhan. (2021). Impact of Visual Impairment on the Wellbeing and Functional Disability of Patients with Glaucoma in India. *Journal of Current Ophthalmology*, *33*(2), 152–157. https://doi.org/10.1016/j.joco.2019.09.006

Sun, M. J., Rubin, G. S., Akpek, E. K., & Ramulu, P. Y. (2017). Impact of Glaucoma and Dry Eye on Text-Based Searching. *Translational Vision Science & Technology*, *6*(3), 24. https://doi.org/10.1167/tvst.6.3.24

Suzuki, S., Murai, H., Imai, T., Nagane, Y., Masuda, M., Tsuda, E., Konno, S., Oji, S., Nakane, S., Motomura, M., Suzuki, N., & Utsugisawa, K. (2014). Quality of life in purely ocular myasthenia in Japan. *BMC Neurology*, *14*(1), 1–6. https://doi.org/10.1186/1471-2377-14-142

Swamy, B., Cumming, R. G., Ivers, R., Clemson, L., Cullen, J., Hayes, M. F., Tanzer, M., & Mitchell, P. (2009). Vision screening for frail older people: A randomised trial. *British Journal of Ophthalmology*, *93*(6), 736–741. https://doi.org/10.1136/bjo.2007.134650

Sweeting, J., Merom, D., Astuti, P. A. S., Antoun, M., Edwards, K., & Ding, D. (2020). Physical activity interventions for adults who are visually impaired: A systematic review and meta-analysis. *BMJ Open*, *10*(2). https://doi.org/10.1136/bmjopen-2019-034036

Szlyk, J. P., Mahler, C. L., Seiple, W., Edward, D. P., & Wilensky, J. T. (2005). Driving performance of glaucoma patients correlates with peripheral visual field loss. *Journal of Glaucoma*, *14*(2), 145–150. https://doi.org/10.1097/01.ijg.0000151686.89162.28

Tagarelli, A., Piro, A., Tagarelli, G., Lantieri, P. B., Risso, D., & Olivieri, R. L. (2004). Colour blindness in everyday life and car driving. *Acta Ophthalmologica Scandinavica*, *82*(4), 436–442. https://doi.org/10.1111/j.1395-3907.2004.00283.x

Tamura, H., Tsukamoto, H., Mukai, S., Kato, T., Minamoto, A., Ohno, Y., Yamashita, H., & Mishima, H. K. (2004). Improvement in cognitive impairment after cataract surgery in elderly patients. *Journal of Cataract and Refractive Surgery*, *30*(3), 598–602. https://doi.org/10.1016/j.jcrs.2003.10.019

Taylor, D. J., Hobby, A. E., Binns, A. M., & Crabb, D. P. (2016). How does age-related macular degeneration affect real-world visual ability and quality of life? A systematic review. *BMJ Open*, *6*(12). https://doi.org/https://doi.org/10.1136/bmjopen-2016-011504

To, K. G., Meuleners, L., Chen, H. Y., Lee, A., van Do, D., van Duong, D., Phi, T. D., Tran, H. H., & Nguyen, N. do. (2014). Assessing the test-retest repeatability of the Vietnamese version of the National Eye Institute 25-item Visual Function Questionnaire among bilateral cataract patients for a Vietnamese population. *Australasian Journal on Ageing*, *33*(2), 8–11. https://doi.org/10.1111/ajag.12129

To, Q., Huynh, V. A., Do, D., Do, V., Congdon, N., Meuleners, L., Vandelanotte, C., Hong, H., Nguyen, H., & To, K. (2021). Falls and Physical Activity among Cataract Patients in Vietnam. *Ophthalmic Epidemiology*, *29*(1), 70–77. https://doi.org/10.1080/09286586.2021.1893341

Travis, L. A., Boerner, K., Reinhardt, J. P., & Horowitz, A. (2004). Exploring functional disability in older adults with low vision. *Journal of Visual Impairment and Blindness*, *98*(9), 534–545. https://doi.org/10.1177/0145482x0409800905

Trousdale, E. R., Hodge, D. O., Baratz, K. H., Maguire, L. J., Bourne, W. M., & Patel, S. v. (2014). Vision-related quality of life before and after keratoplasty for fuchs’ endothelial dystrophy. *Ophthalmology*, *121*(11), 2147–2152. https://doi.org/10.1016/j.ophtha.2014.04.046

Tucha, O., Naumann, M., Berg, D., Alders, G. L., & Lange, K. W. (2001). Quality of life in patients with blepharospasm. *Acta Neurologica Scandinavica*, *103*(1), 49–52. https://doi.org/10.1034/j.1600-0404.2001.00109.x

Uenishi, Y., Tsumura, H., Miki, T., & Shiraki, K. (2003). Quality of life of elderly japanese patients with glaucoma. *International Journal of Nursing Practice*, *9*(1), 18–25. https://doi.org/10.1046/j.1440-172X.2003.00398.x

Van Der Aa, H. P. A., Van Rens, G. H. M. B., Comijs, H. C., Margrain, T. H., Gallindo-Garre, F., Twisk, J. W. R., & Van Nispen, R. M. A. (2015). Stepped care for depression and anxiety in visually impaired older adults: Multicentre randomised controlled trial. *BMJ Open*, *351*. https://doi.org/10.1136/bmj.h6127

van Munster, E. P. J., van der Aa, H. P. A., Verstraten, P., & van Nispen, R. M. A. (2021). Barriers and Facilitators to Identify and Discuss Depression and Anxiety in Visually Impaired Adults : A Qualitative Study on the Service User ’ s Perspective. *BMC Health Services Research*, *21*(1), 1–17. https://doi.org/https://doi.org/10.1186/s12913-021-06682-z

van Nispen, R. M. A., van der Ham, A. L., Hva der Aa, H. P. A., & van Rens, G. H. M. B. (2019). Terugblik  en  toekomstvisie op wetenschap in de visuele sector: Een inventarisatie van onderzoek naar kwaliteit van leven en participatie van mensen met een visuele beperking. *Amsterdam UMC*.

van Nispen, R. M. A., Virgili, G., Hoeben, M., Langelaan, M., Klevering, J., Keunen, J. E. E., & van Rens, G. H. M. B. (2020). Low vision rehabilitation for better quality of life in visually impaired adults. *Cochrane Database of Systematic Reviews*, *2020*(1). https://doi.org/10.1002/14651858.CD006543.pub2

Varma, R., Foong, A. W. P., Lai, M. Y., Choudhury, F., Klein, R., & Azen, S. P. (2010). Four year incidence and progression of age related macular degeneration: The Los Angels Latino Eye Study. *Am J Ophthalmol*, *149*(5), 741–751. https://doi.org/10.1016/j.ajo.2010.01.009.FOUR-YEAR

Vezinaw, C. M., Matchinski, T. L., & Elias, S. (2019). Preferences and Reading Performance of People with Low Vision using a Portable Electronic Magnifier versus a Smartphone Magnification App. *Optometry & Visual Performance*, *7*(1), 53–58. http://bit.ly/2U02Beb

Vin, A., Schneider, S., Muir, K. W., & Rosdahl, J. A. (2015). Health coaching for glaucoma care: A pilot study using mixed methods. *Clinical Ophthalmology*, *9*, 1931–1943. https://doi.org/10.2147/OPTH.S92935

Vu, H. T. V., Keeffe, J. E., McCarty, C. A., & Taylor, H. R. (2005). Impact of unilateral and bilateral vision loss on quality of life. *British Journal of Ophthalmology*, *89*(3), 360–363. https://doi.org/10.1136/bjo.2004.047498

Watkinson, S. (2005). Visual impairment in older people: the nurse’s role. *Nursing Standard (Royal College of Nursing)*, *19*(17), 45–55. https://doi.org/10.7748/ns2005.01.19.17.45.c3782

Whiteside, M. M., Wallhagen, M. I., & Pettengill, E. (2006). Sensory impairment in older adults: Part 2: Vision loss. *American Journal of Nursing*, *106*(11), 52–62. https://doi.org/10.1097/00000446-200611000-00019

White, U. E., Black, A. A., Delbaere, K., & Wood, J. M. (2021). Determinants of concern about falling in adults with age-related macular degeneration. *Ophthalmic and Physiological Optics*, *41*(2), 245–254. https://doi.org/10.1111/opo.12777

William K.S. (2015). *A quantitative study of the relationships between activity limitation and participation restriction among older people with vision impairment and comorbid conditions*. Mississippi State University.

Williams, P. T. (2013). Walking and running are associated with similar reductions in cataract risk. *Medicine and Science in Sports and Exercise*, *45*(6), 1089–1096. https://doi.org/10.1249/MSS.0b013e31828121d0

Willis, J. R., Jefferys, J. L., Vitale, S., & Ramulu, P. Y. (2012). Visual impairment, uncorrected refractive error, and accelerometer-defined physical activity in the United States. *Archives of Ophthalmology*, *130*(3), 329–335. https://doi.org/10.1001/archopthalmol.2011.1773

Wilson, M. R., Coleman, A. L., Yu, F., Fong Sasaki, I., Bing, E. G., & Kim, M. H. (2002). Depression in patients with glaucoma as measured by self-report surveys. *Ophthalmology*, *109*(5), 1018–1022. https://doi.org/10.1016/S0161-6420(02)00993-4

Wittich, W., Murphy, C., & Mulrooney, D. (2014). An adapted adult day centre for older adults with sensory impairment. *British Journal of Visual Impairment*, *32*(3), 249–262. https://doi.org/10.1177/0264619614540162

Wong, S. (2018). Traveling with blindness: A qualitative space-time approach to understanding visual impairment and urban mobility. *Health and Place*, *49*, 85–92. https://doi.org/10.1016/j.healthplace.2017.11.009

Wood, J. M., & Black, A. A. (2016). Ocular disease and driving. *Clinical and Experimental Optometry,* 99(5), 395–401. https://doi.org/10.1111/cxo.12391

Wood, J. M., & Carberry, T. P. (2004). Older drivers and cataracts: Measures of driving performance before and after cataract surgery. *Transportation Research Record*, *1865*(1), 7–13. https://doi.org/10.3141/1865-02

Woods, R. L., & Satgunam, P. (2011). Television, computer and portable display device use by people with central vision impairment. *Ophthalmic and Physiological Optics*, *31*(3), 258–274. https://doi.org/10.1111/j.1475-1313.2011.00833.x

Wrzesińska, M. A., Tabała, K., & Stecz, P. (2021). Gaming behaviors among polish students with visual impairment. *International Journal of Environmental Research and Public Health*, *18*(4), 1–12. https://doi.org/10.3390/ijerph18041545

Xinzhi Zhang, Kai McKeever Bullard, Mary Frances Cotch, M.Roy Wilson, Barry W. Rovner, Gerald McGwin Jr, Cynthia Owsley, Lawrence Barker, John E. Crews, & Jinan B. Saaddine. (2013). Association Between Depression and Functional Vision Loss inPersons 20 Years of Age or Older in the United States, NHANES2005–2008. *JAMA Ophthalmol*, *131*(5), 573–581. https://doi.org/10.1001/jamaophthalmol.2013.2597.Association

Yamanishi, R., Uchino, M., Kawashima, M., Uchino, Y., Yokoi, N., & Tsubota, K. (2019). Characteristics of Individuals with Dry Eye Symptoms without Clinical Diagnosis: Analysis of a Web-Based Survey. *Journal of Clinical Medicine*, *8*(5), 721. https://doi.org/10.3390/jcm8050721

Yanagisawa, M., Kato, S., Kunimatsu, S., Kobayashi, M., & Ochiai, M. (2010). Association between changes in visual acuity and vision-related quality of life in Japanese patients with low vision. *Ophthalmic Research*, *45*(1), 47–52. https://doi.org/10.1159/000316135

Yildiz, E. H., Cohen, E. J., Virdi, A. S., Hammersmith, K. M., Laibson, P. R., & Rapuano, C. J. (2010). Quality of Life in Keratoconus Patients After Penetrating Keratoplasty. *American Journal of Ophthalmology*, *149*(3), 416–422. https://doi.org/10.1016/j.ajo.2009.10.005

Yioti, G., Stefaniotou, M., Ziavrou, I., Kotsis, K., & Hyphantis, T. (2017). Illness Perceptions, Psychiatric Manifestations, and Quality of Life in Patients with Inherited Retinal Dystrophies. *Seminars in Ophthalmology*, *32*(4), 428–437. https://doi.org/10.3109/08820538.2015.1118136

Yokoi, T., Moriyama, M., Hayashi, K., Shimada, N., Tomita, M., Yamamoto, N., Nishikawa, T., & Ohno-Matsui, K. (2014). Predictive factors for comorbid psychiatric disorders and their impact on vision-related quality of life in patients with high myopia. *International Ophthalmology*, *34*(2), 171–183. https://doi.org/10.1007/s10792-013-9805-8

Yoshida, Y., Hiratsuka, Y., Kawachi, I., Murakami, A., Kondo, K., & Aida, J. (2020). Association between visual status and social participation in older Japanese: The JAGES cross-sectional study. *Social Science and Medicine*, *253*. https://doi.org/10.1016/j.socscimed.2020.112959

Zhang, X., Kahende, J., Fan, A. Z., Barker, L., Thompson, T. J., Mokdad, A. H., Li, Y., & Saaddine, J. B. (2011). Smoking and visual impairment among older adults with age-related eye diseases. *Preventing Chronic Disease*, *8*(4).

Zhang, X., Olson, D. J., Le, P., Lin, F. C., Fleischman, D., & Davis, R. M. (2017). The Association Between Glaucoma, Anxiety, and Depression in a Large Population. *American Journal of Ophthalmology*, *183*, 37–41. https://doi.org/10.1016/j.ajo.2017.07.021

Zhanna, B., Magzumova, R., Zeinet, A., & Manshuk, Y. (2018). The effect of age and social status on the quality of life of patients with pathology of the retina and optic nerve. *Drug Invention Today*, *10*(5), 3892–3896.

Zhu, M., Huang, J., Zhu, B., Sun, Q., Xu, X., Miao, Y., & Zou, H. (2015). Changes of vision-related quality of life in retinal detachment patients after cataract surgery. *PLoS ONE*, *10*(3). https://doi.org/10.1371/journal.pone.0120505

Zijlstra, G. A. R., Ballemans, J., & Kempen, G. I. J. M. (2013). Orientation and mobility training for adults with low vision: A new standardized approach. *Clinical Rehabilitation*, *27*(1), 3–18. https://doi.org/10.1177/0269215512445395

Zimdars, A., Nazroo, J., & Gjonça, E. (2012). The circumstances of older people in England with self-reported visual impairment: A secondary analysis of the English Longitudinal Study of Ageing (ELSA). *The British Journal of Visual Impairment*, *30*(1), 22–30. https://doi.org/10.1177/0264619611427374

## Supplementary material: Appendix C: Overview of the included outcome measurements

| Melbourne Low Vision ADL Index (MLVAI) |
| --- |
| COPM |
| NEI - VFQ-39 |
| Visual Status Inventory (VSI) |
| Lowe's visual functioning questionnaire |
| VF-RPR |
| NEI - VFQ-25 |
| ADVIS (activities of daily vision scale) |
| VFQ-48 |
| VAQ (Visual Activities Questionnaire) |
| VDA |
| VPQ (visual performance questionnaire) |
| Functional assessments of low vision (FALV) |
| D-AI |
| AI (Massof) |
| GDS-15 (geriatric depression scale) |
| LVQOL |
| VF-14 |
| VF-11 |
| Functional Independence Measure for Blind Adults (FIMBA) |
| BRSFOutSur |
| Independent Mobility Questionnaire (IMQ) |
| GO-QOL |
| CES-D |
| VROOM (Vision-Related Outcomes in Orientation and Mobility) |
| General health questionnaire (GHQ-28) |
| OMO |
| HADS (Hospital Anxiety and Depression Scale) |
| ADVS (Activities of Daily Living Scale) |
| PAY-YA |
| SF-36 |
| SF-12 |
| modified diabetes quality of life questionnaire (MDQOL) |
| Patient Satisfaction Questionnaire-18 (PSQ-18) |
| Vision Core- Measure 1 (VCM1) |
| Vision and Quality of Life index (VisQoL) |
| Impact of Vision Impairment (IVI) |
| Patient Health Questionnaire-9 (PHQ-9) |
| Participation and Activity Inventory (PAI) |
| Impact on Participation and Autonomy (IPA) |
| Sickness Impact profile (SIP) |
| Macular Disease Dependent Quality of Life (MacDQoL) |
| Medical Outcomes Study short form (MOS) |
| Social activities checklist |
| communicative activiteis checklist |
| daily living tasks dependent on vision questionnaire (DLTV) |
| Glaucoma Quality of Life 15 (GQL-15) |
| WHO-QOL-BREF |
| Impact of Vision Impairment questionnaire (IVI) |
| Low Luminance Questionnaire |
| Sense of well-being inventory (SWBI) |
| the Lubben social network scale (LSNS-6) |
| Glaucoma Symptom Scale (GSS) |
| Ocular Surface Disease Index (OSDI) |
| WHOQOL-100 |
| EQ5D |
| Pittsburgh Sleep Quality Index PSQI |
| Cambridge Glaucoma Visual Function Test |
| GAD (oldberg Anxiety and Depression scale) |
| Tinetti Performance Oriented Mobility Assessment tool |
| Smith’s Activity of Daily Living Independence Survey |
| TED-QOL questionnaire |
| AQoL-7 (incl Vision related QOL (VRQOL)) |
| berdeen Glaucoma Questionnaire (AGQ) |
| daily activities dependence on vision scale (DADV) |
| Glaucoma Activity Limitation 9 (GAL-9) |
| Treatment Impact Patient SatisfactionScale (TIPS) |
| MMSE: blind version |
| The Friendship Scale and the Life Space Questionnaire (LSQ) |
| Hamilton Depression scale (HAM-D) |
| Falls Screening and Refferral Algorithm (FSRA) |
| Orientation and Mobility Test Battery |
| Independent Mobility Questionnaire |
| Barthel index |
| i-CAARV |
| Richman Contrast Sensitivity (SPARCS) tes |
| Indian Visual Functioning Questionnaire (IND-VFQ) |
| Zung Self-Rating Depression Scale |
| Adult Strabismus-20 (AS-20) |
| Assessment of Disability Related to Vision (ADREV) |
| Tarragona Anxiety Questionnaire for the Blind (Cuestionario Tarragona de Ansiedad para Ciegos) (CTAC) |
| SPARCS |
| Lubben Social Network Scale (LSNS-6 |
| LIFE-H (Assessment of Life Habits) |
| driving habit survey |
| Morningness–Eveningness Questionnaire (MEQ) question-naire |
| Sleepiness Scale – ESS |
| NightVision Questionnaire |
| Functional Reading Independence Index (FRII) |
| Impact of Vision Impairment – Very Low Vision (IVI-VLV) questionnaire |
| Hamilton AnxietyRating Scale (HARS) |
| Veterans Affairs Low-Vision Visual Functioning Questionnaire |
| Depression Anxiety Stress Scale (DASS-21) |
| Kessler-10 psycho-logical distress scale (K-10) |
| Comprehensive Psycho-pathological Rating Scale (CPRS |
| Beck Depression Inventory-II(BDI-II |
| Catquest-9SF |
| Falls EfficacyScale–International (FES-I) |
| Athlete Satisfaction Questionnaire (ASQ) |
| Graves’ Ophthalmopathy QOL instrument(GO-QOL) |
